# Dynamic selection for forage quality and quantity in response to phenology and insects in an Arctic ungulate

**DOI:** 10.1002/ece3.7852

**Published:** 2021-08-04

**Authors:** Heather E. Johnson, Trevor S. Golden, Layne G. Adams, David D. Gustine, Elizabeth A. Lenart, Perry S. Barboza

**Affiliations:** ^1^ Alaska Science Center U.S. Geological Survey Anchorage Alaska; ^2^ U.S. Fish and Wildlife Service Anchorage Alaska; ^3^ Alaska Department of Fish and Game Fairbanks Alaska; ^4^ Texas A & M University College Station Texas; ^5^ Present address: Axiom Data Science 1016 West 6th Avenue Anchorage Alaska 99501

**Keywords:** Alaska, biomass, caribou, digestible energy, digestible nitrogen, *Rangifer tarandus*

## Abstract

Spatiotemporal variation in forage is a primary driver of ungulate behavior, yet little is known about the nutritional components they select, and how selection varies across the growing season with changes in forage quality and quantity. We addressed these uncertainties in barren‐ground caribou (*Rangifer tarandus*), which experience their most important foraging opportunities during the short Arctic summer. Recent declines in Arctic caribou populations have raised concerns about the influence of climate change on summer foraging opportunities, given shifting vegetation conditions and insect harassment, and their potential effects on caribou body condition and demography. We examined Arctic caribou selection of summer forage by pairing locations from females in the Central Arctic Herd of Alaska with spatiotemporal predictions of biomass, digestible nitrogen (DN), and digestible energy (DE). We then assessed selection for these nutritional components across the growing season at landscape and patch scales, and determined whether foraging opportunities were constrained by insect harassment. During early summer, at the landscape scale, caribou selected for intermediate biomass and high DN and DE, following expectations of the forage maturation hypothesis. At the patch scale, however, caribou selected for high values of all forage components, particularly DN, suggesting that protein may be limiting. During late summer, after DN declined below the threshold for protein gain, caribou exhibited a switch at both spatial scales, selecting for higher biomass, likely enabling mass and fat deposition. Mosquito activity strongly altered caribou selection of forage and increased their movement rates, while oestrid fly activity had little influence. Our results demonstrate that early and late summer periods afford Arctic caribou distinct foraging opportunities, as they prioritize quality earlier in the summer and quantity later. Climate change may further constrain caribou access to DN as earlier, warmer Arctic summers may be associated with reduced DN and increased mosquito harassment.

## Graphical abstract

We examined the summer foraging behavior of Arctic caribou to assess selection for different nutritional components across the growing season at landscape and patch scales, and determine whether foraging opportunities were constrained by insect harassment. We found that caribou selected for forage quality early in the summer likely enabling protein deposition, while they selected for forage quantity later in the summer likely enabling fat deposition. Mosquito harassment strongly altered caribou selection of forage and increased their energy expenditure, while oestrid fly harassment had relatively little influence.

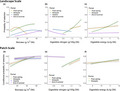

## 1 INTRODUCTION

The behavior and demography of ungulate populations are largely driven by bottom‐up forage conditions (Fritz & Duncan, 1994; Sinclair & Krebs, 2002; White, 1983), as variation in forage influences ungulate activities and movement rates (Coulombe et al., 2008; Wickstrom et al., 1984), patterns of migration and habitat use (Zweifel‐Schielly et al., 2009; Merkle et al., 2016), nutritional condition (Proffitt et al., 2016), demographic rates (Cook et al., 2004), and the trajectories of populations (Messier et al., 1988). Although seasonal variation in the foodscape underpins ungulate ecology (Searle et al., 2007), difficulties in quantifying forage conditions at landscape scales have precluded our understanding of the nutritional components selected by ungulates, and how their foraging decisions vary across the year and at different spatial scales (Felton et al., 2018). Addressing these uncertainties is becoming increasingly important as climate change alters the phenology, composition, biomass, and nutrient content of plants (Lin et al., 2010; Parmesan & Yohe, 2003; Yuan & Chen, 2015) with anticipated impacts on the behavior and demography of ungulate populations (Mallory & Boyce, 2017; Weiskopf et al., 2019).

Ungulates are generally expected to select forage based on trade‐offs between quantity and quality (Fryxell, 1991). Forage quality (i.e., digestible energy and digestible protein) tends to be high in new plant growth at the start of the growing season when forage quantity is low, and then declines as plants mature and their defensive tissues and compounds increase (Hebblewhite et al., 2008; Van Soest, 1982). The forage maturation hypothesis predicts that ungulates should take advantage of this trade‐off, selecting forage in earlier phenological stages with intermediate biomass to maximize their consumption of digestible nutrients (Fryxell, 1991). Field studies have corroborated this hypothesis as ungulates have been observed to select for intermediate forage biomass and earlier plant phenology (Hebblewhite et al., 2008; Raynor et al., 2016), particularly at the start of the growing season when they follow the “green wave” of new vegetation along elevational and latitudinal gradients (Merkle et al., 2016; Aikens et al., 2017). While the forage maturation hypothesis has been useful for making predictions about ungulate behavior under spring conditions, little is known about how selection shifts later in the growing season after forage quality declines, despite the importance of late summer foraging for accruing body fat and increasing reproductive success (Cook et al., 2004; Tollefson et al., 2010). Additionally, selection for forage quantity–quality trade‐offs can be scale dependent (Hebblewhite et al., 2008; Wilmshurst et al., 1999), with ungulates generally expected to prioritize selection for coarser factors, such as biomass, at larger spatial scales, while prioritizing finer factors, such as forage quality, at smaller spatial scales (Bailey et al., 1996). Some studies provide support for this expectation (Van Beest et al., 2010; Wilmshurst et al., 1999), but others have found that selection remains consistent across scales (Zweifel‐Schielly et al., 2009) or exhibits more nuanced patterns (Balluffi‐Fry et al., 2020; St‐Louis & Côté, 2014), confounding our understanding of the hierarchical processes driving foraging behavior.

While forage quantity is commonly estimated as biomass, there is significant uncertainty about what constitutes forage quality for many ungulates. It is often assumed that quality is determined by the digestible energy content of plants (Fryxell, 1991; Hebblewhite et al., 2008; Parker et al., 2009), although evidence suggests that digestible protein (i.e., nitrogen) may also be critical (Albon & Langvatn, 1992; McArt et al., 2009; Zweifel‐Schielly et al., 2009). Energy enables body maintenance, growth, and the deposition of fat reserves, while protein is required for maintenance, growth, and reproduction (Parker et al., 2009). Variation in the relative availability of these two forage components may determine which one is nutritionally limiting, and thus, driving patterns of selection (Berteaux et al., 1998). For example, digestible nitrogen may be preferred early in the growing season when it is abundant in immature plants (Albon & Langvatn, 1992; Zweifel‐Schielly et al., 2009) particularly in nitrogen‐poor environments (Cain et al., 2017; McArt et al., 2009). Meanwhile, digestible energy may be selected more strongly later in the summer as animals store fat for the coming winter (Cook et al., 2004; Parker et al., 2009). Unfortunately, past studies of foraging behavior have often assessed ungulate selection for either digestible energy or protein, but not both, contributing to confusion about their relative influence in ungulate habitat selection, and how selection may change across the growing season and at different spatial scales (Felton et al., 2018).

**FIGURE 1:**
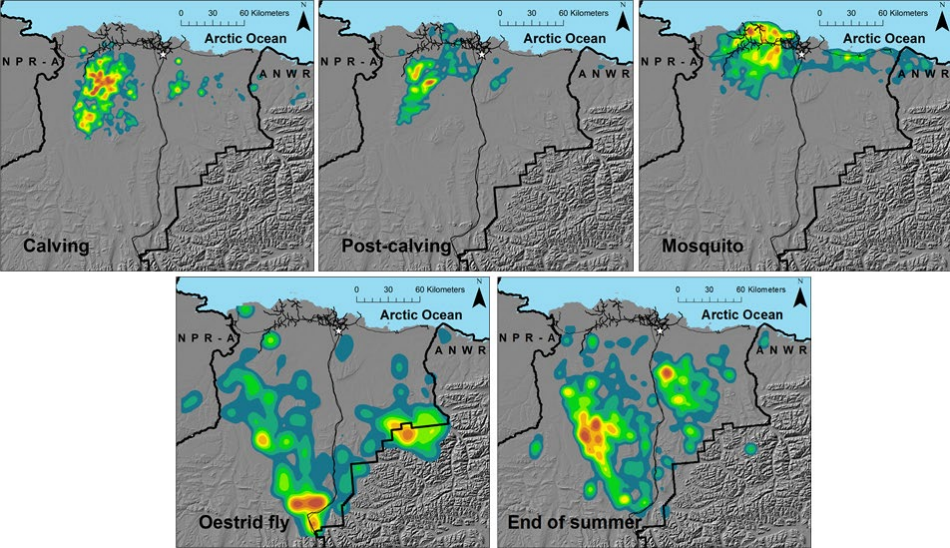
Density plots of Central Arctic Herd female caribou locations during the calving (1–15 June), post‐calving (16–24 June), mosquito harassment (25 June–15 July), oestrid fly harassment (29 July–7 August), and end of summer (16–31 August) periods on the North Slope of Alaska, 2015–2018. Warmer colors indicate greater densities of caribou locations, thin black lines are roads and pipelines associated with energy development, and the white star is Deadhorse, Alaska. The study area is primarily managed by the State of Alaska (unmarked lands), the U.S. Fish and Wildlife Service Arctic National Wildlife Refuge (ANWR), and the Bureau of Land Management National Petroleum Reserve—Alaska (NPR‐A; ownership boundaries are in thick black lines)

Spatiotemporal variation in forage conditions is particularly important in driving the behavior and dynamics of migratory, barren‐ground caribou (*Rangifer tarandus*; Crête & Huot, 1993; Messier et al., 1988; Schaefer et al., 2016), which experience their greatest foraging opportunities during the short Arctic summer (Parker et al., 2009). Recent declines in several Arctic herds have raised concerns about the influence of warming Arctic conditions on summer range quality and the subsequent effects on caribou demography (Fauchald et al., 2017; Mallory & Boyce, 2017). Given the limited period when high quality forage is available in the Arctic, investigators have speculated that even small reductions in summer foraging opportunities could have cascading effects on caribou body condition, and subsequently, demographic rates (White, 1983). Arctic caribou exhibit some of the longest migrations of any terrestrial mammal on earth (Joly et al., 2020) to reach summer ranges where they birth calves, regain body stores lost during the previous winter, and amass reserves for the upcoming winter and reproductive cycle (Barboza & Parker, 2008; Taillon et al., 2013; White et al., 1975). In addition to supplying important forage resources, summer ranges also provide caribou with insect relief habitat and reduced predator abundance (Griffith et al., 2002; White et al., 1975). Unlike ungulates in temperate systems, Arctic caribou typically migrate through snow, arriving at their calving grounds prior to the onset of vegetation green‐up (Gurarie et al., 2019; Laforge et al., 2021). Once on their summer ranges, Arctic caribou often exhibit dynamic patterns of habitat use, shifting their distributions and habitat selection patterns every few weeks (Wilson et al., 2012; Bureau of Land Management [BLM], Bureau of Land Mangement, 2019a; Johnson et al., 2020; Severson et al., 2021; Figure 1). Researchers have assumed that their behavior is influenced, in part, by spatiotemporal variation in the nutritional value of forage (Cameron et al., 2005; Griffith et al., 2002), but the underlying drivers remain unknown.

Given the challenges of quantifying caribou forage conditions across expansive, remote Arctic landscapes, past studies on the summer foraging behavior of caribou and conspecific reindeer are limited and have yielded mixed results. Like other ungulates, caribou and reindeer should select forage in accordance with the forage maturation hypothesis, but summer research on reindeer at finer scales has found that they prioritize forage quantity over quality (Van der Wal et al., 2000; Mårrell et al., 2002), although they have also been observed to broadly track the receding snow to consume highly digestible plants (Skogland, 1980). Although energy is typically considered the main currency of forage quality in ungulates, Barboza et al. (2018) hypothesized that Arctic caribou were instead limited by protein, given that, as capital breeders, they must amass protein during summer to reproduce the following year (Barboza & Parker, 2008; Taillon et al., 2013). Barboza et al. (2018) found that Arctic caribou were only able to store protein reserves during the early part of the summer, when digestible quantities exceeded maintenance levels, in contrast to digestible energy, which remained high in most forage plants throughout the summer. These mixed reports on the importance of different forage components for caribou have made it difficult to infer their likely responses to future environmental conditions. Warmer temperatures and earlier phenology in the Arctic are increasing the biomass (Doiron et al., 2014; Elmendorf et al., 2012), but potentially reducing the quality (Johnson et al., 2018; Zamin et al., 2017) of summer forage, while also altering the period it is available (Gustine et al., 2017). Our ability to predict how changing climate conditions may impact the summer foraging opportunities of caribou relies upon a clear understanding of which forage components are selected, which components are limiting, and how selection changes across the growing season at different spatial scales.

As warmer, longer growing seasons are altering Arctic forage conditions, they may also be influencing the ability of caribou to consume forage due to extended, more intense periods of insect harassment (Culler et al., 2015). During mid‐summer, when temperatures peak, caribou and reindeer exhibit strong behavioral responses to mosquitoes (*Culicidae*) and oestrid flies (*Oestridae*, also known as warble flies and nasal botflies) as they decrease foraging, increase walking and running, and move to cooler, windier areas to avoid harassment (Hagemoen & Reimers, 2002; White et al., 1975; Witter, Johnson, Croft, Gunn, & Gillingham, 2012). Years with increased harassment have been associated with reduced fall weights in reindeer (Helle & Tarvainen, 1984; Weladji et al., 2003) likely due to a combination of lower forage intake and higher energy expenditure. Although insect harassment is well recognized to influence summer caribou behavior, it remains unclear whether the intensity of harassment alters foraging behavior via scaled or threshold responses, and how responses may vary for different insect types. Because caribou–insect interactions will change with warming Arctic weather (Witter, Johnson, Croft, Gunn, & Poirier, 2012), insect harassment has the potential to constrain caribou foraging opportunities in the future.

While investigators commonly examine ungulate foraging behavior by correlating their movements to indices of plant phenology (Merkle et al., 2016; Aikens et al., 2017), we examined caribou behavior as a function of the specific nutritional components of forage, capitalizing on recent models by Johnson et al. (2018). These models used field data on the quantity and quality of important caribou forages throughout the growing season, in conjunction with nonlinear relationships with the Normalized Difference Vegetation Index (NDVI) and other habitat characteristics, to predict weekly spatiotemporal variation in biomass, digestible nitrogen (DN) and digestible energy (DE) across the summer range of the Central Arctic Herd (CAH) in northern Alaska. We paired these predictive forage models with locations of GPS‐collared CAH females to test several hypotheses about the nutritional factors selected by caribou across the growing season at both landscape and patch scales, and whether selection was influenced by insect harassment. Examining selection at the landscape scale enabled us to assess factors driving shifts in caribou distributions within their summer range, while examining selection at the patch scale enabled us to assess fine‐scale foraging decisions.

**FIGURE 2:**
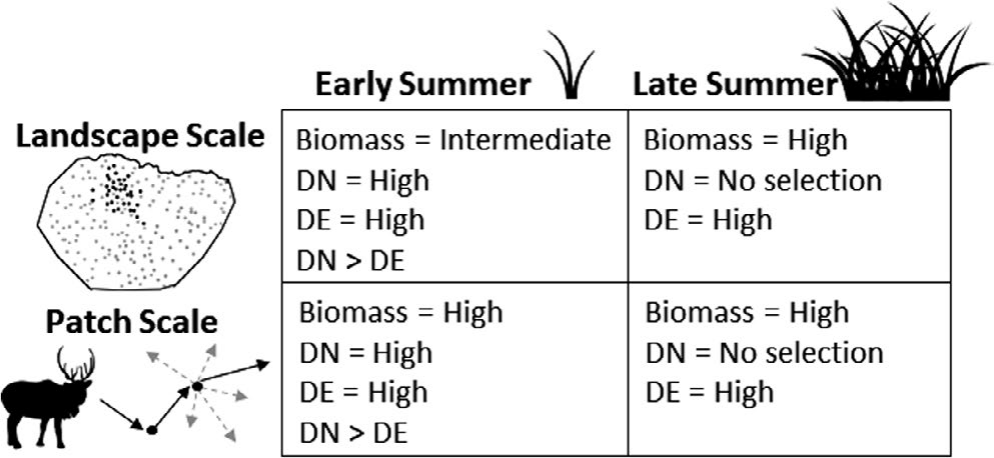
Predictions of how Arctic caribou select for forage biomass, digestible nitrogen (DN), and digestible energy (DE) during early and late summer at landscape and patch scales

We expected selection of summer forage quantity and quality by Arctic caribou to be highly dynamic relative to the period of the growing season, the spatial scale investigated, and the intensity of insect harassment. During the early summer, at the landscape scale, we hypothesized that caribou would select nutritional components in accordance with the forage maturation hypothesis, in that they would select for intermediate forage biomass and high forage quality (Figure 2). At the patch scale however, we predicted that caribou would select for high values of all forage components, including biomass, reflecting fine‐scale observations of reindeer (Mårrell et al., 2002; Van der Wal et al., 2000). We also tested the hypothesis that forage DN was limiting and predicted that caribou would select more strongly for DN than DE at both spatial scales during the early summer when DN was abundant (Barboza et al., 2018; Figure 2). During late summer, at both spatial scales, we hypothesized that caribou would prioritize fat deposition by selecting areas with higher values of forage biomass and DE (Chan‐McLeod et al., 1994), but exhibit no selection for DN, as it would have declined below expected thresholds for storage (Figure 2). Finally, during the insect periods, we hypothesized that greater harassment would reduce caribou selection for preferred forage components and increase their movement rates, factors which both constrain foraging opportunities. Testing this suite of hypotheses enabled us to elucidate the dynamic roles of forage and insect conditions on shaping summer caribou behavior, with key implications for how a warming Arctic may alter foraging opportunities for caribou in the future.

## 2 METHODS

### 2.1 Study system

The CAH summer range includes portions of the Arctic Coastal Plain, and the foothills and mountains of the Brooks Range, Alaska, USA (Figure 1). The Arctic Coastal Plain gradually rises from sea level along the Arctic Ocean to ~250 m. It is largely covered by thaw lakes and wetland complexes dominated by wet graminoid vegetation that includes water sedge (*Carex aquatilis*) and tall cottongrass (*Eriophorum angustifolium*). The foothills continue to rise in elevation and primarily consist of a treeless band of plateaus and hills covered by tussock cottongrass (*Eriophorum vaginatum*), Bigelow's sedge (*Carex bigelowii*), diamond‐leaf willow (*Salix pulchra*), and dwarf birch (*Betula nana*) in the uplands, and water sedges in the lowlands. Mountain habitats used by the CAH during summer rise to ~1,000 m and consist of slopes sparsely covered with dwarf shrub vegetation (willow [*Salix* spp.], dwarf birch) and mesic valleys dominated by graminoids. Summers in this region are characterized as short, cool, and moist, while winters are long, cold, and dry with annual precipitation averaging 103 mm (Deadhorse weather station; http://climate.gi.alaska.edu/Climate/Normals). Between 2010 and 2018, the average temperature in July (warmest month) was 9.2℃ and in February (coolest month) was −23.3℃ (http://climate.gi.alaska.edu/acis_data). Lands used by CAH during summer are primarily managed by the State of Alaska, North Slope Borough, and the U.S. Fish and Wildlife Service (Figure 1).

In May, female CAH caribou migrate north from the Brooks Range to their calving grounds on the Arctic Coastal Plain. After calving in early June, they continue to move north toward the coast during the mid‐summer period of mosquito harassment where the weather is cooler and windier (White et al., 1975), and then shift south toward the foothills later in the summer (Figure 1). In the fall, CAH caribou typically migrate to winter ranges on the south side of the Brooks Range, but sometimes remain on the Arctic Coastal Plain (Nicholson et al., 2016). The herd reached peak abundance in 2010 at ~68,000 caribou but subsequently declined, and was estimated at ~30,000 in 2019 (Alaska Department of Fish and Game [ADFG], 2020).

### 2.2 Caribou location data

During 2015–2018, we captured female CAH caribou (≥2 years old) via net‐gun following protocols approved by ADFG (2015‐06, 2016‐30, 0019‐2017‐19, and 0019‐2018‐49). Captures were conducted in April 2015 and 2017, and in June 2016 and 2018. Caribou were fit with GPS satellite collars (Telonics Inc., Mesa, Arizona) programmed to collect a location every 2 hr (GPS collar data managed by ADFG). For animals captured in June, we excluded locations from the first week those animals were collared to reduce any capture‐related effects. During the study, we collected data from 71 individual caribou and obtained a total of 140 animal‐year data sets (16 in 2015, 28 in 2016, 44 in 2017, and 52 in 2018). We separated caribou locations into 5 periods recognized by management agencies as characterizing distinct behaviors (Person et al., 2007; Wilson et al., 2012): calving (1 June–15 June), post‐calving (16 June–24 June), mosquito harassment (25 June–15 July), oestrid fly harassment (29 July–7 August), and end of summer (16–31 August). Gaps in time before the oestrid fly and end of summer periods excluded days when caribou were typically moving between habitat areas, so that we estimated selection during periods when caribou behavior was relatively consistent. We considered “early summer” to be the calving, post‐calving, and mosquito harassment periods, and “late summer” to be the oestrid fly harassment and end of summer periods.

### 2.3 Characterizing forage, habitat, and insect conditions

We used approaches and models in Johnson et al. (2018) to predict forage biomass, DN, and DE (250 m resolution) each week during 1 June–31 August with data from Adams and Gustine (2018). To briefly summarize that work, Johnson et al. (2018) used NDVI values from eMODIS ALASKA data (Jenkerson et al., 2010), along with vegetation type (Boggs et al., 2016) and distance to the coast, to model weekly variation in summer field measurements of the quantity and quality of 6 key summer forage species for caribou on the North Slope of Alaska (Griffith et al., 2002; Russell et al., 1993; Thompson & McCourt, 1981; White & Trudell, 1980): tussock cottongrass, water sedge, Bigelow's sedge, Arctic lousewort (*Pedicularis* spp.), diamond‐leaf willow, and Richardson's willow (*Salix richardsonii*). Forage data were collected at 9 macroplots within the CAH summer range every 2 weeks, June–August, 2011–2013 (Gustine et al., 2017). At each site, during each sampling occasion, aboveground biomass of the current annual growth of each forage species was clipped, dried, and weighed. Representative samples of each forage species were analyzed for digestible nitrogen and energy (Johnson et al., 2018; Van Someren et al., 2015), which were averaged across the species present on the plot. Johnson et al. (2018) modeled biomass (g/m^2^), digestible nitrogen (g/m^2^), and digestible energy (kJ/m^2^) per unit area (m^2^) of forage. We used their same biomass model, but then adopted their approach to model digestible contents of nitrogen (g/100 g) and energy (kJ/g) in dry mass (DM) of forage. This allowed us to better distinguish forage abundance (biomass) from forage quality (concentration). Details of our DN and DE models are provided in Appendix S1.

We then used those forage models, along with 2015–2018 NDVI data and spatial data on vegetation type and distance to the coast, to generate weekly raster predictions of biomass (g/m^2^ DM), DN (g/100 g DM), and DE (kJ/g DM) across the CAH summer range. Only positive NDVI values that were scored as “good quality” (Jenkerson et al., 2010) were used for modeling (i.e., poor quality, cloud, and snow values were considered missing data), with values scaled between 0 and 1. If a forage component (biomass, DN or DE) in a raster pixel was predicted to be <0, it was assigned a 0 value. During summer, forage that provides <1 g N/100 g DM or <9 kJ/g DM is likely to impair female caribou, as below these thresholds they are unable to compensate for poor forage quality with increased intake (Barboza & Parker, 2008; Barboza et al., 2009; Chan‐McLeod et al., 1994).

While our primary objective was to investigate caribou selection of forage conditions, we also accounted for topography and snowmelt characteristics that have previously been shown to be important in the selection of summer habitat by Arctic caribou (Baltensperger & Joly, 2019; Johnson et al., 2020). We created rasters depicting elevation (m) and aspect (categorical: north, east, south, west, and flat) from the U.S. Geological Survey National Elevation Dataset (http://www.usgs.gov) that were summarized at a 250 m pixel resolution that matched the spatial scale of our forage predictions. We also used rasters depicting the last day of snow (ordinal day, hereafter “snowmelt date”) from the Geographic Network of Alaska which used MODIS snow data (http://www.gina.alaska.edu/projects/modis‐derived‐snow‐metrics; 500 m resolution).

We indexed mosquito and oestrid fly activity at caribou locations using equations from Russell et al. (1993, 2013), as these indices have been highly effective at predicting insect‐driven shifts in Arctic caribou behavior (Cameron et al., 1995; Wilson et al., 2012; Prichard et al., 2020). The mosquito index (MI) and oestrid fly index (OI) were based on temperature and wind speed conditions specific to each insect, ranging from 0 to 1, where higher values indicated greater insect activity. Both indices were 1 when the temperature was ≥18⁰C and the wind speed was 0 m/s, while the MI was 0 when the temperature was <6⁰C or the wind speed was >6 m/s, and the OI was 0 when the temperature was <13⁰C or the wind speed was >9 m/s. The MI was derived from field data on mosquito activity, but the OI was based on a literature review, as oestrid flies are difficult to sample in the field (Russell et al., 1993). During the mosquito and oestrid fly periods, MI and OI were calculated for each caribou location, respectively, based on the spatial coordinates, date, and time using spatial hourly temperature and wind speed data from the Modern‐Era Retrospective Analysis for Research and Applications Version 2 (https://gmao.gsfc.nasa.gov/reanalysis/MERRA‐2/; resolution 0.625°×0.5°).

### 2.4 Statistical analyses

#### 2.4.1 Landscape scale forage selection

We tested our hypotheses about caribou selection for forage quantity and quality at a landscape scale using resource selection function analyses (Manly et al., 2002), quantifying population‐level patterns of selection across the summer range (second order selection, individual selection with the population summer range; Meyer & Thuiller, 2006) using a use‐availability design. We delineated available habitat for all periods as the 100% minimum convex polygon around summer caribou locations (1 June–31 August), removing areas that overlapped with the Arctic Ocean. Because vegetation type was included as a covariate within our forage models (Johnson et al., 2018), we only retained caribou locations located within the 4 vegetation types that defined our forage prediction area (tussock tundra, herbaceous mesic, herbaceous wet, or dwarf shrub; Boggs et al., 2016), which covered 73% of the vegetation in the summer range and overlapped 84% of caribou locations. Missing vegetation types included low shrub (16%), bare ground/sparse vegetation (5%), herbaceous marsh (3%), fire scar (2%), and tall shrub (1%). Low and tall shrubs were dispersed throughout the central and southern portions of the summer range, while marsh vegetation was dispersed along the coastal plain, and the fire scar was located in the southwest portion of the study area. For each used location, we randomly generated 10 “available” locations within the forage prediction area with the same timestamp. Used and available locations were attributed with their spatiotemporally matched predicted forage values (biomass, DN, and DE), habitat characteristics (elevation, snowmelt date, and aspect), and insect activity indices (MI and OI) during their respective periods (see Appendix S2 for variable means and ranges).

We conducted resource selection analyses using generalized linear mixed models (Bolker et al., 2009) with a logit‐link function to accommodate our use‐availability design via the “lme4” package (Bates et al., 2015) in R version 3.6.1 (R Core Team, 2019). For each summer period, we included a random effect for each animal‐year data set and scaled continuous variables (all variables except aspect) to facilitate model convergence and the interpretation of relative effects (Schielzeth, 2010). To account for general trends in habitat use, we included aspect, elevation, and snowmelt date in all our models, along with quadratic terms for elevation and snowmelt date. We ensured that multicollinearity was not an issue among covariates by assessing correlation coefficients (*r* ≤ |0.7|; Dormann et al., 2013).

To evaluate support for our predictions about how caribou would select forage quantity and quality at the landscape scale (Figure 2), we tested a series of models. For each early and late summer period, we tested a null model, a base habitat model (that included aspect, elevation, elevation^2^, snowmelt date, and snowmelt date^2^), and the base habitat model in addition to all subsets of our forage components (biomass, DN, and DE). We tested linear relationships with DN and DE and linear and quadratic relationships with biomass, given predictions from the forage maturation hypothesis. During the oestrid fly period, biomass and DE were negatively correlated (*r* = −0.83) so we tested models with biomass separately from those with DE. We identified the best‐supported model for each period based on Akaike's Information Criterion (AIC) and model weights (Burnham & Anderson, 2002). Additionally, we assessed whether insect harassment reduced caribou selection for forage components. Using our top forage models for the mosquito and oestrid fly periods, we tested whether the inclusion of MI or OI within their respective periods, and interactions between the insect indices and forage components, significantly improved model fit.

We used k‐fold cross‐validation (Boyce et al., 2002) to assess the fit of our top forage and insect models, including all locations from each animal‐year data set in either the model training or testing set. We used fivefolds and 10 bins, and repeated the process 10 times to generate a mean Spearman correlation for each top model.

#### 2.4.2 Patch scale forage selection

For each early and late summer period, we modeled caribou selection for forage components at the patch scale (fourth order selection, individual site selection within a habitat patch; Meyer & Thuiller, 2006) using step‐selection analyses (Fortin et al., 2005; Thurfjell et al., 2014). We identified caribou “steps” as consecutive fixes collected 2 hr apart. Because our forage predictions were constrained to specific vegetation types, we retained only those steps that ended within our prediction area. For each caribou step, we randomly generated 10 available steps from the same starting location. Available steps were drawn from period‐specific parametric distributions of step length (Forester et al., 2009) and a uniform distribution of turning angles (Panzacchi et al., 2016). If the end location of an available step landed in the Arctic Ocean or outside the forage prediction area, we re‐generated the step. Used and available steps were attributed with the predicted forage values (biomass, DN, and DE), habitat characteristics, and insect indices (during the corresponding insect season) of their endpoints, along with step length (see Appendix S2 for variable means and ranges).

For each summer period, we analyzed the attributes of used and available steps with conditional logistic regression, using the clogit function in the R “survival” package (Therneau, 2015). We included a step identifier as the strata and clustered by animal‐year to account for the lack of independence between steps made by the same animal within a summer (Prima et al., 2017). Additionally, to reduce bias in coefficient estimates we included step length (m) in all models (Forester et al., 2009) and calculated robust standard errors. As in the landscape scale analyses, all models included the habitat variables of aspect, elevation, and snowmelt date, with quadradic terms for elevation and snowmelt date. We standardized continuous variables and ensured that variables were not highly correlated. We used the Quasi‐likelihood Independence Criterion (QIC; Pan, 2001) to evaluate relative support for different models (Craiu et al., 2008), as QIC can be used to compare the parsimony of models fit to autocorrelated data.

Similar to our landscape scale analyses, we assessed support for hypotheses about caribou selection for forage quantity and quality at the patch scale (Figure 2) by testing a series of models. We tested a null model, a base habitat model (including the same habitat variables as previously described), and the base habitat model in addition to all subsets of our forage variables. During the oestrid fly period, biomass and DE were negatively correlated (*r* ≤ −0.85) so we did not include those variables in the same models. To evaluate whether insect harassment reduced caribou selection of forage quantity or quality during the mosquito and oestrid fly periods, we tested whether the inclusion of MI or OI (within their respective periods) in our top forage models, and interactions between insect indices and forage components, improved model fit. We used k‐fold cross‐validation to assess the fit of all top forage and insect models, using the approach previously described.

#### 2.4.3 Movement rates

We tested whether caribou increased their movement rates in response to mosquito and oestrid fly harassment, by using linear mixed models to determine whether observed step lengths increased as a function of the insect indices. In our models, we log transformed step lengths to normalize their distributions and included animal‐year as a random effect. We also included ordinal day as a nuisance parameter, since the movement rates of Arctic caribou exhibit distinct patterns across the summer (Person et al., 2007). We tested models with linear and quadratic terms for the insect indices and ordinal day, and used AIC values and model weights to identify the top performing model for each insect period.

## 3 RESULTS

**FIGURE 3:**
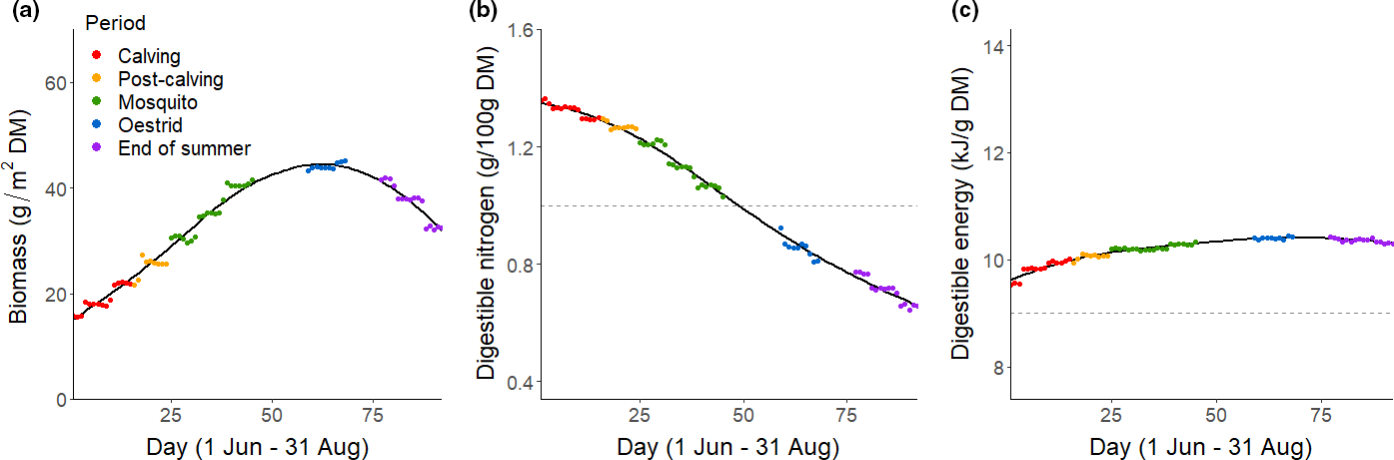
Predicted daily average values of forage (a) biomass, (b) digestible nitrogen, and (c) digestible energy at available caribou locations across the summer (1 June–31 August; days 1–92) at the landscape scale for the Central Arctic Caribou Herd, Alaska, 2015–2018. Forage values are color coded by whether they were predicted to occur during the calving (red; 1–15 June), post‐calving (orange; 16–24 June), mosquito harassment (green; 25 June–15 July), oestrid fly harassment (blue; 29 July–7 August), or end of summer (purple; 16–31 August) periods. The black lines depict a smoothed model fit to the average daily forage values, and the horizontal dotted reference lines on the digestible nitrogen (1g N/100g dry mass [DM]) and energy (9 kJ/g DM) plots indicate likely thresholds for protein and energy gain in caribou, respectively. The y‐axes are scaled to show 95% of the range of predicted forage values

Average predicted forage values were highly variable across the growing season, particularly for biomass and DN (Figure 3). Throughout the study area, forage biomass increased during early and mid‐summer, reaching a peak in early August before starting to decline (Figure 3a). Meanwhile, DN was highest at the start of the growing season and declined across the summer, dropping below the threshold likely needed to store protein in mid‐July (Figure 3b). Average predicted DE exhibited an increase during early summer, and then was relatively consistent throughout the remainder of the summer (Figure 3c), remaining well above the threshold estimated for mass gain.

We were unable to predict forage values for caribou locations and steps when NDVI values were categorized as snow, clouds, or having poor data quality. At the landscape scale, this resulted in missing forage values for 46%, 13%, 8%, 8%, and 27% of calving, post‐calving, mosquito, oestrid fly, and end of summer caribou locations, respectively, while at the patch scale, this resulted in missing values for 60%, 13%, 1%, 6%, and 20% of steps, respectively. The particularly high proportion of missing forage values during the calving period was the result of persistent snow cover on the summer range during early June (Appendix S3). As a result, we did not assess caribou selection for forage components during this period. The increase in missing values during the end of summer was due to greater cloud cover.

We analyzed 110 animal‐year datasets collected during the post‐calving period, 140 during the mosquito harassment period, 131 during the oestrid fly harassment period, and 128 during the end of summer period. For landscape scale analyses, we employed a total of 7,510, 20,725, 7,011, and 12,501 used caribou locations during the post‐calving, mosquito, oestrid fly, and end of summer periods, respectively. For patch scale analyses, we employed 7,422, 20,293, 6,867, and 12,232 used steps during those periods, respectively.

### 3.1 Landscape scale forage selection

**FIGURE 4:**
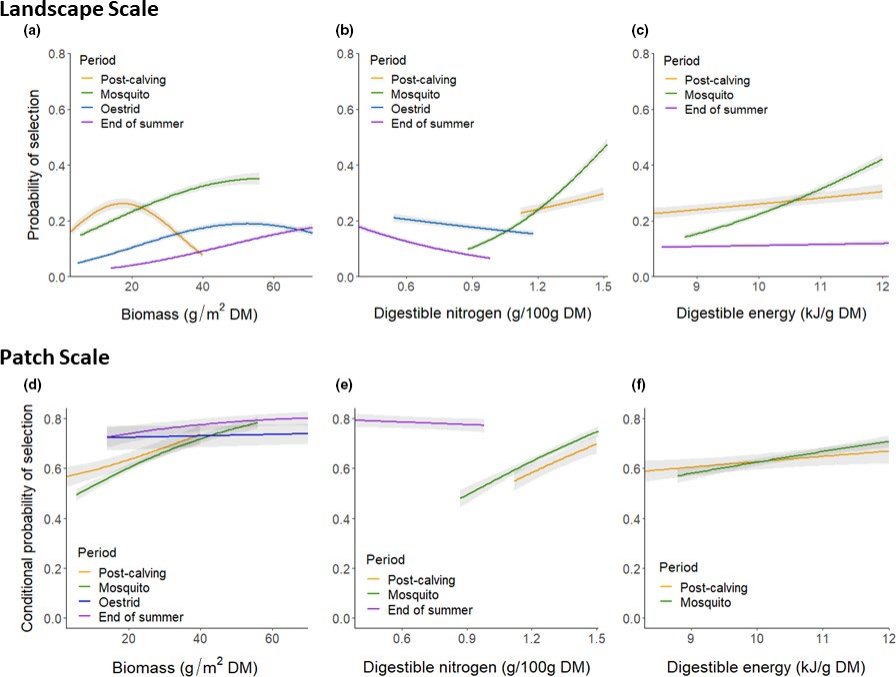
Relative probabilities of selection (and 95% confidence intervals) of female caribou for forage biomass, digestible nitrogen, and digestible energy at landscape (a‐c) and patch (d‐f) scales during the post‐calving (16–24 June), mosquito harassment (25 June–15 July), oestrid fly harassment (29 July–7 August), and end of summer (16–31 August) periods, Central Arctic Herd, Alaska, 2015–2018. Probabilities are only shown for variables included in the top forage model for each scale and period and displayed for 95% of the range of forage values for used locations while holding all other covariates at their mean values for used locations

At the landscape scale, all forage variables (including the quadratic term for biomass) were included in the top models for all periods, except the oestrid fly period when only biomass and DN were included (but due to collinearity biomass and DE were not tested within the same models). The top model for each period received ≥87% of the model weight (Table 1). During the early summer, caribou selected for intermediate biomass and high DN and DE during the post‐calving period as predicted (Figure 4a–c; Table 2), but they selected for high biomass, DN, and DE during the mosquito period (Figure 4a–c; Table 2; but see additional Results below). During the late summer, caribou selected areas with intermediate‐high biomass and lower DN during the oestrid fly period, and for high biomass and DE (although selection for DE was weak) but low DN during the end of summer (Figure 4a–c; Table 2). Generally, at the landscape scale, caribou selected forage components most strongly during the early summer. Although we expected caribou to exhibit greater selection for DN than DE during the early summer, selection for these components of forage quality was equal (Table 2). Caribou selection for forage during the first summer period (post‐calving) and last summer period (end of summer) generally aligned with our early and late summer predictions, respectively (Figure 2), while the intermediate summer periods (mosquito and oestrid fly harassment) exhibited more mixed results.

**TABLE 1:** Landscape scale model selection criteria for forage models including biomass (BM; g/m^2^ dry mass [DM]), digestible nitrogen (DN; g/100 g DM), and digestible energy (DE; kJ/g DM) selected by female caribou in the Central Arctic Herd during the post‐calving (16–24 June), mosquito harassment (25 June–15 July), oestrid fly harassment (29 July–7 August), and end of summer (16–31 August) periods, Alaska, 2015–2018

Period and Model	LL	AIC	ΔAIC	Weight
Post‐calving				
Base habitat + BM + BM^2^ + DN + DE	−20,205.49	40,438.98	0.00	1.00
Base habitat + BM + BM^2^ + DN	−20,221.40	40,468.80	29.82	0.00
Base habitat + BM + BM^2^ + DE	−20,225.64	40,477.28	38.31	0.00
Base habitat + BM + BM^2^	−20,229.80	40,483.61	44.63	0.00
Base habitat + BM + DN + DE	−20,719.88	41,465.76	1,026.79	0.00
Base habitat + BM + DN	−20,731.53	41,487.05	1,048.07	0.00
Base habitat + BM	−20,748.42	41,518.83	1,079.86	0.00
Base habitat + BM + DE	−20,747.50	41,519.01	1,080.03	0.00
Base habitat + DN + DE	−20,859.00	41,742.00	1,303.02	0.00
Base habitat + DE	−21,025.87	42,073.74	1634.77	0.00
Base habitat + DN	−21,078.25	42,178.50	1739.52	0.00
Base habitat	−21,160.34	42,340.68	1901.70	0.00
Null	−31,067.1	62,138.20	21,699.22	0.00
Mosquito harassment				
Base habitat + BM + BM^2^ + DN + DE	−49,397.90	98,823.79	0.00	1.00
Base habitat + BM + DN + DE	−49,502.55	99,031.09	207.30	0.00
Base habitat + DN + DE	−49,701.56	99,427.12	603.33	0.00
Base habitat + BM + BM^2^ + DN	−49,791.79	99,609.57	785.78	0.00
Base habitat + BM + DN	−49,934.16	99,892.33	1,068.54	0.00
Base habitat + DN	−49,955.98	99,933.97	1,110.17	0.00
Base habitat + BM + BM^2^ + DE	−50,017.75	100,061.51	1,237.72	0.00
Base habitat + BM + BM^2^	−50,030.63	100,085.25	1,261.46	0.00
Base habitat + BM + DE	−50,181.99	100,387.97	1564.18	0.00
Base habitat + BM	−50,198.07	100,418.14	1594.35	0.00
Base habitat + DE	−50,224.51	100,471.01	1647.22	0.00
Base habitat	−50,324.02	100,668.03	1844.24	0.00
Null	−78,738.40	157,480.80	58,657.01	0.00
Oestrid fly harassment				
Base habitat + BM + BM^2^ + DN	−20,891.92	41,809.84	0.00	1.00
Base habitat + BM + BM^2^	−20,925.60	41,875.20	65.36	0.00
Base habitat + BM + DN	−21,100.28	42,224.57	414.73	0.00
Base habitat + BM	−21,117.30	42,256.50	446.66	0.00
Base habitat + DN + DE	−21,299.10	42,622.20	812.36	0.00
Base habitat + DE	−21,304.40	42,630.90	821.06	0.00
Base habitat + DN	−21,352.00	42,726.00	916.16	0.00
Base habitat	−21,389.30	42,798.60	988.76	0.00
Null	−25,832.60	51,669.30	9,859.46	0.00
End of summer				
Base habitat + BM + BM^2^ + DN + DE	−38,378.68	76,785.36	0.00	0.87
Base habitat + BM + BM^2^ + DN	−38,381.54	76,789.08	3.72	0.13
Base habitat + BM + DN	−38,452.32	76,928.65	143.29	0.00
Base habitat + BM + DN + DE	−38,452.18	76,930.35	145.00	0.00
Base habitat + BM + BM^2^ + DE	−38,640.77	77,307.53	522.18	0.00
Base habitat + BM + DN	−38,676.68	77,377.35	591.99	0.00
Base habitat + BM + BM^2^	−38,691.85	77,407.71	622.35	0.00
Base habitat + BM	−38,737.65	77,497.29	711.94	0.00
Base habitat + DN + DE	−38,944.17	77,912.35	1,126.99	0.00
Base habitat + DE	−38,958.31	77,938.61	1,153.26	0.00
Base habitat + DN	−39,367.46	78,756.92	1971.57	0.00
Base habitat	−39,439.10	78,898.19	2,112.83	0.00
Null	−53,183.60	106,371.20	29,585.84	0.00

Notes

“Base habitat” for all periods included aspect, elevation, elevation^2^, snowmelt date, and snowmelt date^2^. During the oestrid fly harassment period, biomass and DE were negatively correlated; so those forage components were not included in the same models.

**TABLE 2:** Landscape scale standardized coefficients from top forage models of female caribou selection during the post‐calving (16–24 June), mosquito harassment (25 June–15 July), oestrid fly harassment (29 July–7 August), and end of summer (16–31 August) periods in the Central Arctic Herd, Alaska, 2015–2018

Model and Coefficient	β	SE	*p*	L95% CI	U95% CI
Post‐calving					
Intercept	−10.497	0.357	<0.001	−11.196	−9.798
Biomass	1.534	0.074	<0.001	1.390	1.679
Biomass^2^	−2.643	0.097	<0.001	−2.833	−2.453
Digestible nitrogen	0.153	0.024	<0.001	0.105	0.201
Digestible energy	0.155	0.028	<0.001	0.101	0.209
Elevation	2.178	0.217	<0.001	1.752	2.604
Elevation^2^	−20.673	1.026	<0.001	−22.683	−18.663
Snowmelt date	2.109	0.238	<0.001	1.643	2.575
Snowmelt date^2^	−2.084	0.236	<0.001	−2.547	−1.620
Aspect (reference = North)					
East	0.355	0.049	<0.001	0.259	0.450
South	0.505	0.054	<0.001	0.398	0.611
West	−0.001	0.049	0.982	−0.097	0.095
Flat	−0.390	0.042	<0.001	−0.472	−0.309
Mosquito harassment					
Intercept	−4.970	0.200	<0.001	−5.362	−4.578
Biomass	0.943	0.043	<0.001	0.860	1.027
Biomass^2^	−0.617	0.044	<0.001	−0.704	−0.531
Digestible nitrogen	0.660	0.020	<0.001	0.622	0.699
Digestible energy	0.660	0.024	<0.001	0.613	0.708
Elevation	−5.824	0.129	<0.001	−6.077	−5.570
Elevation^2^	1.744	0.569	0.002	0.628	2.859
Snowmelt date	−2.117	0.108	<0.001	−2.328	−1.905
Snowmelt date^2^	2.160	0.107	<0.001	1.951	2.369
Aspect (reference = North)					
East	0.139	0.036	<0.001	0.069	0.209
South	−0.059	0.045	0.192	−0.147	0.029
West	−0.167	0.035	<0.001	−0.236	−0.097
Flat	−0.415	0.028	<0.001	−0.471	−0.360
Oestrid fly harassment					
Intercept	−2.608	0.036	<0.001	−2.679	−2.537
Biomass	1.392	0.055	<0.001	1.284	1.501
Biomass^2^	−1.079	0.055	<0.001	−1.187	−0.971
DN	−0.112	0.013	<0.001	−0.138	−0.085
Elevation	1.349	0.026	<0.001	1.299	1.400
Elevation^2^	−0.215	0.011	<0.001	−0.238	−0.193
Snowmelt date	0.054	0.017	0.002	0.020	0.088
Snowmelt date^2^	−0.023	0.008	0.004	−0.038	−0.007
Aspect (reference = North)					
East	0.010	0.041	0.805	−0.070	0.090
South	0.299	0.044	<0.001	0.213	0.385
West	0.063	0.038	0.096	−0.011	0.138
Flat	0.413	0.049	<0.001	0.317	0.508
End of summer					
Intercept	−2.762	0.031	<0.001	−2.823	−2.702
Biomass	1.199	0.056	<0.001	1.088	1.309
Biomass^2^	−0.504	0.043	<0.001	−0.588	−0.420
Digestible nitrogen	−0.360	0.016	<0.001	−0.391	−0.328
Digestible energy	0.055	0.023	0.018	0.010	0.101
Elevation	0.938	0.048	<0.001	0.069	0.199
Elevation^2^	−1.115	0.068	<0.001	−0.077	0.078
Snowmelt date	0.050	0.149	0.740	0.157	0.276
Snowmelt date^2^	−0.131	0.149	0.378	0.495	0.614
Aspect (reference = North)					
East	0.134	0.033	<0.001	0.843	1.033
South	0.000	0.040	0.996	−1.248	−0.983
West	0.217	0.030	<0.001	−0.243	0.342
Flat	0.554	0.031	<0.001	−0.423	0.160

Note

The forage variables included biomass (g/m^2^ dry mass [DM]), digestible nitrogen (g/100 g DM), and digestible energy (kJ/g DM).

**FIGURE 5:**
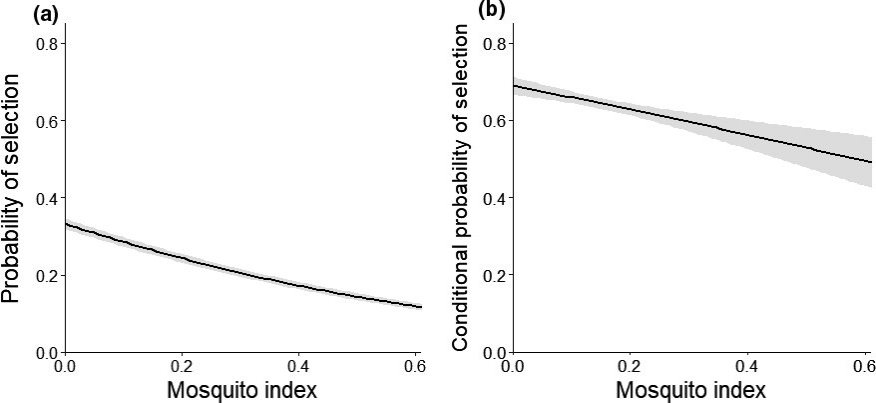
Relative probabilities of selection (and 95% confidence intervals) of female caribou for areas with different levels of the mosquito index at the (a) landscape and (b) patch scale during the mosquito harassment period (25 June–15 July), Central Arctic Herd, Alaska, 2015–2018. Probabilities were derived from the top forage model for each scale while holding all other covariates at their mean values for used locations

**FIGURE 6:**
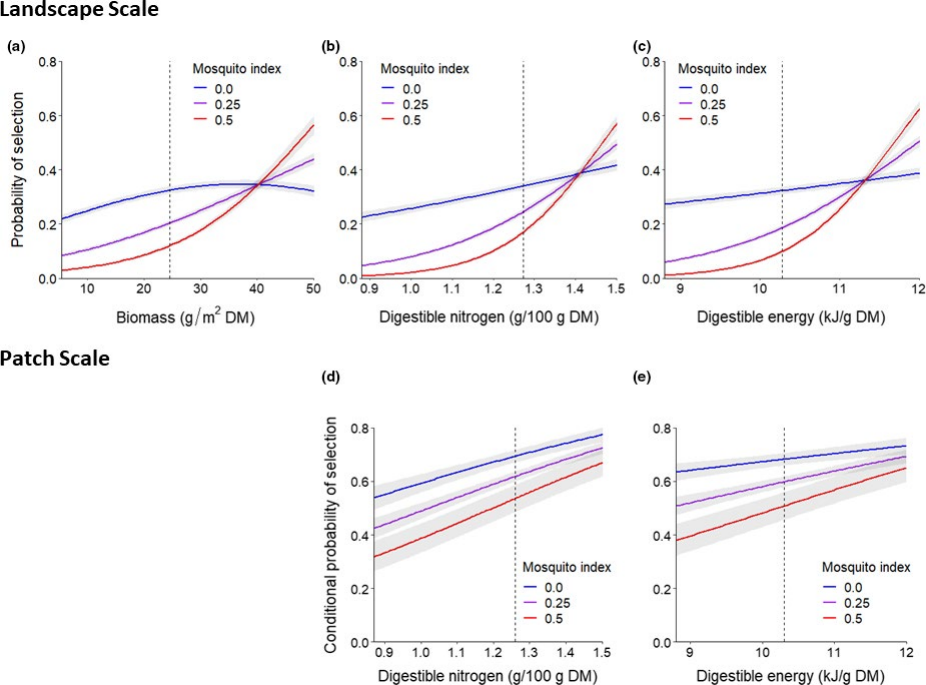
Relative probabilities of selection (and 95% confidence intervals) of female caribou for (a) biomass, (b) digestible nitrogen, and (c) digestible energy at the landscape scale and conditional relative probabilities of selection for (d) digestible nitrogen and (e) digestible energy at the patch scale under different levels of the mosquito index during the mosquito harassment period (25 June–15 July), Central Arctic Herd, Alaska, 2015–2018. At the patch scale, an interaction between biomass and the mosquito index is not shown because it was excluded from the top model. Model effects are displayed for 95% of the range of forage values for used locations while holding all other covariates at their mean values for used locations. The dotted reference lines depict the median value of each forage component at used locations

We found strong evidence that the index of mosquito activity altered caribou selection for forage at the landscape scale (Table 3). During the mosquito harassment period, the inclusion of MI and interactions between MI and all forage components significantly improved model fit, obtaining 100% of the model weight (Table 3). Caribou selected habitat with lower MI values (Figure 5a; Table 4). Interestingly, when the MI was low, caribou selected areas with intermediate forage biomass as we predicted (Figure 2; Figure 6a), but when the MI increased, caribou selected for greater biomass. This switch appeared to be driven by their increased use of herbaceous mesic vegetation, reduced use of herbaceous wet vegetation, and greater use of areas with advanced phenology, preferences which all likely reduced their exposure to mosquitos. Higher MI values generally depressed caribou selection for DN and DE, except when DN and DE were exceedingly high and strongly selected (Figure 6b, c). It is important to emphasize that these results are relevant only within our forage prediction area, which excluded bare ground and sparse vegetation often used by CAH when harassed by insects (White et al., 1975).

**TABLE 3:** Landscape‐scale model selection criteria for models testing the inclusion of insect indices (mosquito index = MI, oestrid fly index = OI), and interactions between insect indices and forage components, to the top forage model for the mosquito (25 June–15 July) and oestrid fly (29 July–7 August) harassment periods, respectively. The forage model for the mosquito harassment period included biomass (BM; g/m^2^ dry mass [DM]), biomass^2^, digestible nitrogen (DN; g/100 g DM), digestible energy (DE; kJ/g DM), aspect, elevation, elevation^2^, snowmelt date, and snowmelt date^2^. The forage model for the oestrid fly harassment period included all of the same variables except for DE. Selection models are for female caribou from the Central Arctic Herd, Alaska, 2015–2018

Period and Model	LL	AIC	ΔAIC	Weight
Mosquito harassment				
Forage model + MI + MI*BM + MI*BM^2^ + MI*DN + MI*DE	−48,050.64	96,139.29	0.00	1.00
Forage model + MI + MI*DN + MI*DE	−48,405.42	96,844.85	705.56	0.00
Forage model + MI + MI*BM + MI*BM^2^ + MI*DE	−48,500.83	97,037.66	898.37	0.00
Forage model + MI + MI*BM + MI*BM^2^ + MI*DN	−48,544.19	97,124.38	985.09	0.00
Forage model + MI + MI*DE	−48,547.47	97,126.95	987.66	0.00
Forage model + MI + MI*DN	−48,591.08	97,214.16	1,074.87	0.00
Forage model + MI + MI*BM + MI*BM^2^	−48,618.40	97,270.80	1,131.51	0.00
Forage model + MI	−48,691.81	97,413.62	1,274.33	0.00
Forage model	−49,397.90	98,823.79	2,684.50	0.00
Oestrid fly harassment				
Forage model + OI + OI*BM + OI*BM^2^ + OI*DN	−20,757.70	41,549.40	0.00	0.52
Forage model + OI + OI*BM + OI*BM^2^	−20,758.78	41,549.55	0.15	0.48
Forage model + OI	−20,887.14	41,802.27	252.87	0.00
Forage model + OI + OI*DN	−20,887.14	41,804.27	254.87	0.00
Forage model	−20,891.92	41,809.84	260.44	0.00

**TABLE 4:** Landscape scale standardized coefficients from top forage*insect index (mosquito index = MI, oestrid fly index = OI) interaction models of female caribou habitat selection during the mosquito harassment (25 June–15 July) and oestrid fly harassment (29 July–7 August) periods in the Central Arctic Herd, Alaska, 2015–2018

Model and Coefficient	β	SE	*p*	L95% CI	U95% CI
Mosquito harassment					
Intercept	−5.564	0.184	<0.001	−5.925	−5.203
MI	−0.596	0.015	<0.001	−0.626	−0.567
Biomass	1.220	0.056	<0.001	1.110	1.330
Biomass^2^	−0.260	0.050	<0.001	−0.359	−0.162
Digestible nitrogen	1.043	0.024	<0.001	0.996	1.090
Digestible energy	1.358	0.035	<0.001	1.291	1.426
MI*Biomass	0.242	0.054	<0.001	0.136	0.347
MI*Biomass^2^	0.562	0.050	<0.001	0.465	0.659
MI*Digestible nitrogen	0.629	0.022	<0.001	0.587	0.672
MI*Digestible energy	0.941	0.031	<0.001	0.880	1.001
Elevation	−5.032	0.127	<0.001	−5.281	−4.783
Elevation^2^	−0.052	0.528	0.921	−1.086	0.982
Snowmelt date	−2.080	0.108	<0.001	−2.292	−1.868
Snowmelt date^2^	2.106	0.107	<0.001	1.897	2.315
Aspect (reference = North)					
East	0.150	0.036	<0.001	0.079	0.220
South	−0.023	0.046	0.620	−0.112	0.067
West	−0.144	0.036	<0.001	−0.213	−0.074
Flat	−0.376	0.029	<0.001	−0.432	−0.319
Oestrid fly harassment					
Intercept	−2.350	0.073	<0.001	−2.494	−2.206
OI	0.125	0.057	0.029	0.013	0.237
Biomass	1.404	0.056	<0.001	1.294	1.514
Biomass^2^	−1.082	0.056	<0.001	−1.191	−0.973
Digestible nitrogen	−0.585	0.075	<0.001	−0.732	−0.439
OI*Biomass	−0.367	0.048	<0.001	−0.461	−0.272
OI*Biomass^2^	0.183	0.053	0.001	0.080	0.286
OI*Digestible nitrogen	−0.098	0.067	0.142	−0.230	0.033
Elevation	1.769	0.046	<0.001	1.679	1.860
Elevation^2^	−0.752	0.042	<0.001	−0.834	−0.670
Snowmelt date	0.506	0.163	0.002	0.187	0.825
Snowmelt date^2^	−0.479	0.162	0.003	−0.797	−0.162
Aspect (reference = North)					
East	0.017	0.041	0.683	−0.064	0.097
South	0.296	0.044	<0.001	0.210	0.383
West	0.060	0.038	0.115	−0.015	0.135
Flat	0.411	0.049	<0.001	0.315	0.506

Note

The forage variables included biomass (g/m^2^ dry mass [DM]), digestible nitrogen (g/100g DM), and digestible energy (kJ/g DM).

**FIGURE 7:**
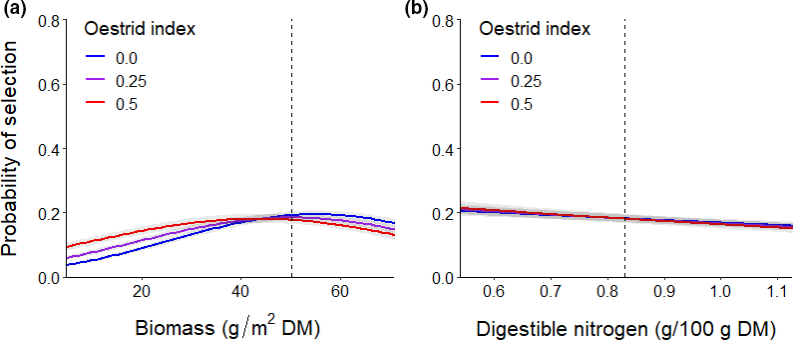
Landscape‐scale probabilities of selection (and 95% confidence intervals) of female caribou for forage (a) biomass and (b) digestible nitrogen under different levels of oestrid fly activity during the oestrid fly harassment period (29 July–7 August), Central Arctic Herd, Alaska, 2015–2018. Model effects are displayed for 95% of the range of forage values for used locations while holding all other covariates at their mean values for used locations. The dotted reference lines depict the median value of each forage component at used locations

Oestrid fly activity also influenced caribou selection for forage (Table 3), but the effects were weak (Table 4). The top forage*OI model had 52% of the weight and included OI and interactions between OI and biomass and DN (Table 3), although the confidence interval for the coefficient for the OI*DN interaction overlapped zero (Table 4). The second best model, with the remaining 48% of the weight, excluded this interaction. Generally, caribou did not alter their selection of habitat based on the OI alone (Appendix S4), but the OI interacted with the forage components such that caribou experiencing greater fly activity were more likely to select areas with less biomass (Figure 7a) and select more strongly for areas with lower DN (Figure 7b).

All landscape scale top forage and insect models exhibited high predictive power based on k‐fold cross‐validation. The mean *r_s_* for the post‐calving, mosquito, oestrid fly, and end of summer models were 0.97, 0.99, 0.91, and 0.96, respectively. The mean *r_s_* for the top forage*mosquito and forage*oestrid fly models were 0.98 and 0.94, respectively.

### 3.2 Patch scale forage selection

At the patch scale, caribou exhibited strong selection for forage during the early summer, exhibiting behaviors that aligned with our predictions (Figure 2). The top models during the post‐calving and mosquito periods indicated that caribou selected for higher values of all forage components (model weights ≥0.87%; Tables 5 and 6; Figure 4d–f), with selection for DN almost twice as strong as DE (Table 6). Selection of forage during the late summer periods, however, exhibited greater model uncertainty. During the oestrid fly period, the top forage model included only selection for biomass (Table 5) but the addition of this term did not significantly improve the fit of the base habitat model (reduced QIC by only 1.17). During the end of summer, the addition of forage components to the base habitat model improved fit (Table 5), but besides the linear term for biomass, there was uncertainty about which additional forage variables should be included. The top model garnered only 18% of the model weight and included biomass (linear and quadratic terms) and DN. During both late summer periods, caribou selected for higher biomass as predicted (although the effects were relatively weak), but showed no selection for DE (Figure 4d, f). We predicted that caribou would exhibit no selection for DN during late summer. While this expectation was met during the oestrid fly period (Table 5), during the end of summer caribou selected areas with lower DN than what was available (Figure 4e), although the CIs for the coefficient of DN overlapped zero (Table 6).

**TABLE 5:** Patch scale model selection criteria for forage models including biomass (BM; g/m^2^ dry mass [DM]), digestible nitrogen (DN; g/100g DM), and digestible energy (DE; kJ/g DM) selected by female caribou in the Central Arctic Herd during the post‐calving (16–24 June), mosquito harassment (25 June–15 July), oestrid fly harassment (29 July–7 August), and end of summer (16–31 August) periods, Alaska, 2015–2018

Period and Model	LL	QIC	ΔQIC	Weight
Post‐calving				
Base habitat + BM + BM^2^ + DN + DE	−15,573.39	31,206.49	0.00	0.87
Base habitat + BM + DN + DE	−15,576.01	31,210.59	4.09	0.11
Base habitat + BM + BM^2^ + DN	−15,579.27	31,214.16	7.67	0.02
Base habitat + BM + DN	−15,582.08	31,218.31	11.82	0.00
Base habitat + BM	−15,607.59	31,267.62	61.13	0.00
Base habitat + BM + BM^2^	−15,606.78	31,267.85	61.36	0.00
Base habitat + BM + BM^2^ + DE	−15,605.87	31,269.46	62.97	0.00
Base habitat + BM + DE	−15,606.84	31,269.83	63.33	0.00
Base habitat + DN	−15,614.17	31,280.48	73.99	0.00
Base habitat + DN + DE	−15,611.75	31,280.61	74.12	0.00
Base habitat + DE	−15,617.59	31,289.81	83.32	0.00
Base habitat	−15,623.51	31,296.83	90.34	0.00
Null	−15,675.92	31,383.42	176.93	0.00
Mosquito harassment				
Base habitat + BM + BM^2^ + DN + DE	−43,039.84	86,122.75	0.00	1.00
Base habitat + BM + DN + DE	−43,046.36	86,133.63	10.88	0.00
Base habitat + BM + BM^2^ + DN	−43,071.74	86,183.18	60.43	0.00
Base habitat + BM + DN	−43,078.11	86,193.86	71.12	0.00
Base habitat + BM + BM^2^ + DE	−43,132.75	86,305.85	183.10	0.00
Base habitat + BM + BM^2^	−43,139.31	86,316.31	193.56	0.00
Base habitat + BM + DE	−43,141.07	86,320.59	197.85	0.00
Base habitat + BM	−43,148.22	86,332.30	209.56	0.00
Base habitat + DN + DE	−43,186.22	86,410.60	287.85	0.00
Base habitat + DE	−43,190.06	86,416.14	293.39	0.00
Base habitat + DN	−43,248.26	86,531.46	408.71	0.00
Base habitat	−43,281.06	86,595.29	472.54	0.00
Null	−43,398.07	86,809.50	686.75	0.00
Oestrid fly harassment				
Base habitat + BM	−14,786.93	29,624.08	0.00	0.36
Base habitat	−14,789.01	29,625.25	1.17	0.20
Base habitat + BM + DN	−14,786.87	29,626.04	1.96	0.13
Base habitat + DE	−14,788.40	29,626.08	2.00	0.13
Base habitat + DN	−14,788.89	29,627.12	3.04	0.08
Base habitat + DN + DE	−14,788.21	29,627.81	3.73	0.06
Base habitat + BM + BM^2^	−14,786.91	29,628.89	4.81	0.03
Base habitat + BM + BM^2^ + DN	−14,786.85	29,630.87	6.79	0.01
Null	−14,881.58	29,781.38	157.30	0.00
End of summer				
Base habitat + BM + BM^2^ + DN	−23,570.82	47,195.93	0.00	0.18
Base habitat + BM + DE	−23,572.70	47,196.08	0.14	0.16
Base habitat + BM + DN	−23,572.58	47,196.41	0.47	0.14
Base habitat + BM	−23,574.00	47,196.50	0.57	0.13
Base habitat + BM + BM^2^	−23,572.67	47,196.70	0.77	0.12
Base habitat + BM + DN + DE	−23,571.88	47,197.20	1.27	0.09
Base habitat + BM + BM^2^ + DE	−23,571.97	47,197.44	1.51	0.08
Base habitat + BM + BM^2^ + DN + DE	−23,570.66	47,197.93	1.99	0.06
Base habitat + DE	−23,575.52	47,199.92	3.98	0.02
Base habitat + DN + DE	−23,575.48	47,202.51	6.58	0.01
Base habitat	−23,588.43	47,222.10	26.16	0.00
Base habitat + DN	−23,588.42	47,224.81	28.87	0.00
Null	−23,611.21	47,236.92	40.99	0.00

Note

“Base habitat” for all periods included aspect, elevation, elevation^2^, snowmelt date, snowmelt date^2^, and step length. During the oestrid fly harassment period, biomass and DE were negatively correlated; so those forage components were not included in the same models.

**TABLE 6:** Patch scale standardized coefficients from top forage models of female caribou selection during the post‐calving (16–24 June), mosquito harassment (25 June–15 July), oestrid fly harassment (29 July–7 August), and end of summer (16–31 August) periods in the Central Arctic Herd, Alaska, 2015–2018

Model and Coefficient	β	Robust SE	*p*	L95% CI	U95% CI
Post‐calving					
Biomass	0.109	0.037	0.003	0.036	0.182
Biomass^2^	0.085	0.033	0.011	0.020	0.151
Digestible nitrogen	0.169	0.027	<0.001	0.117	0.221
Digestible energy	0.085	0.036	0.020	0.013	0.156
Elevation	−0.950	0.118	<0.001	−1.182	−0.718
Elevation^2^	0.049	0.007	<0.001	0.036	0.062
Snowmelt date	−0.015	0.034	0.656	−0.081	0.051
Snowmelt date^2^	−0.004	0.010	0.693	−0.023	0.016
Aspect (reference = North)					
East	−0.008	0.058	0.894	−0.120	0.105
South	0.096	0.070	0.173	−0.042	0.234
West	−0.017	0.053	0.746	−0.121	0.087
Flat	−0.098	0.049	0.046	−0.195	−0.002
Step length	−1.134	0.109	<0.001	−1.347	−0.921
Mosquito harassment					
Biomass	0.476	0.038	<0.001	0.401	0.551
Biomass^2^	−0.116	0.035	0.001	−0.185	−0.047
Digestible nitrogen	0.280	0.026	<0.001	0.230	0.330
Digestible energy	0.161	0.026	<0.001	0.110	0.212
Elevation	−0.490	0.084	<0.001	−0.655	−0.325
Elevation^2^	0.110	0.032	<0.001	0.048	0.172
Snowmelt date	−0.233	0.122	0.055	−0.472	0.005
Snowmelt date^2^	0.214	0.121	0.077	−0.023	0.452
Aspect (reference = North)					
East	−0.008	0.036	0.816	−0.078	0.062
South	−0.031	0.055	0.576	−0.139	0.077
West	−0.186	0.034	<0.001	−0.253	−0.119
Flat	−0.211	0.030	<0.001	−0.271	−0.152
Step length	−0.927	0.037	<0.001	−0.999	−0.855
Oestrid fly harassment					
Digestible energy	0.045	0.027	0.093	−0.007	0.097
Elevation	2.980	0.495	<0.001	2.010	3.949
Elevation^2^	−1.460	0.345	<0.001	−2.135	−0.785
Snowmelt date	0.467	0.383	0.223	−0.285	1.218
Snowmelt date^2^	−0.487	0.384	0.205	−1.240	0.266
Aspect (reference = North)					
East	−0.016	0.052	0.759	−0.117	0.085
South	0.011	0.053	0.838	−0.093	0.115
West	−0.033	0.053	0.532	−0.136	0.070
Flat	−0.017	0.070	0.805	−0.155	0.121
Step length	−1.141	0.086	<0.001	−1.308	−0.973
End of summer					
Biomass	0.228	0.079	0.004	0.074	0.382
Biomass^2^	−0.119	0.072	0.100	−0.261	0.023
Digestible nitrogen	−0.032	0.020	0.111	−0.072	0.007
Elevation	−1.173	0.403	0.004	−1.963	−0.383
Elevation^2^	0.680	0.226	0.003	0.237	1.123
Snowmelt date	0.234	0.269	0.383	−0.293	0.761
Snowmelt date^2^	−0.244	0.266	0.358	−0.765	0.277
Aspect (reference = North)					
East	0.018	0.042	0.668	−0.065	0.102
South	−0.100	0.056	0.073	−0.209	0.010
West	−0.074	0.051	0.150	−0.174	0.027
Flat	−0.025	0.034	0.473	−0.092	0.043
Step length	−2.231	0.086	<0.001	−2.401	−2.062

Note

The forage variables included biomass (g/m^2^ dry mass [DM]), digestible nitrogen (g/100g DM), and digestible energy (kJ/g DM).

At the patch scale, there was evidence that greater mosquito activity reduced caribou selection for DN and DE (Tables 7 and 8). The top model included MI and interactions between MI and DN and DE with 73% of the model weight (Table 7). Caribou were more likely to select areas where the MI was low (Figure 5b), and the strength of their selection for DN and DE declined as the MI increased (Figure 6d, e; Table 8). During the oestrid fly period, we found that the inclusion of OI did not improve the fit of the base habitat model (Table 7). Because neither forage variables nor OI significantly improved the fit of the base habitat model, we did not assess additional forage*OI interactions.

**TABLE 7:** Patch scale model selection criteria for models testing the inclusion of the mosquito index (MI), and interactions between MI and forage components, to the top forage model for the mosquito harassment period (25 June–15 July), and for testing the inclusion of the oestrid fly index (OI) to the base habitat model for the oestrid fly harassment period (29 July–7 August). The forage model for the mosquito harassment period included biomass (BM; g/m^2^ dry mass [DM]), biomass^2^, digestible nitrogen (DN; g/100 g DM), digestible energy (DE; kJ/g DM), aspect, elevation, elevation^2^, snowmelt date, snowmelt date^2^, and step length. The base habitat model was used for the oestrid fly period because forage variables did not significantly improve model fit, and included aspect, elevation, elevation^2^, snowmelt date, snowmelt date^2^, and step length. Selection models are for female caribou from the Central Arctic Herd, Alaska, 2015–2018

Period and Model	LL	QIC	ΔQIC	Weight
Mosquito harassment				
Forage model + MI + MI*DN + MI*DE	−43,007.85	86,065.84	0.00	0.73
Forage model + MI + MI*BM + MI*BM^2^ + MI*DN + MI*DE	−43,007.64	86,069.06	3.22	0.15
Forage model + MI + MI*DE	−43,011.45	86,070.76	4.92	0.06
Forage model + MI + MI*BM + MI*BM^2^ + MI*DE	−43,009.74	86,071.01	5.17	0.06
Forage model + MI + MI*BM + MI*BM^2^	−43,013.45	86,076.49	10.65	0.00
Forage model + MI + MI*BM + MI*BM^2^ + MI*DN	−43,012.99	86,077.51	11.67	0.00
Forage model + MI	−43,027.50	86,100.81	34.97	0.00
Forage model + MI + MI*DN	−43,027.41	86,102.75	36.91	0.00
Forage model	−43,039.84	86,122.75	56.91	0.00
Oestrid fly harassment				
Base habitat model	−14,789.01	29,596.02	0.00	0.50
Base habitat model + OI	−14,788.16	29,596.32	0.03	0.50

**TABLE 8:** Patch scale standardized coefficients from the top forage*mosquito index (MI) interaction model of female caribou habitat selection during the mosquito harassment period (25 June–15 July) in the Central Arctic Herd, Alaska, 2015–2018

Coefficient	β	Robust SE	*p*	L95% CI	U95% CI
MI	−0.2466	0.054	<0.001	−0.3521	−0.1411
Biomass	0.4591	0.039	<0.001	0.3833	0.5348
Biomass^2^	−0.0788	0.035	0.026	−0.1483	−0.0094
Digestible nitrogen	0.2865	0.027	<0.001	0.2341	0.3388
Digestible energy	0.1711	0.027	<0.001	0.1188	0.2234
MI*Digestible nitrogen	0.0351	0.013	0.006	0.0102	0.0601
MI*Digestible energy	0.0648	0.010	<0.001	0.0449	0.0846
Elevation	−0.4538	0.087	<0.001	−0.6234	−0.2842
Elevation^2^	0.0920	0.033	0.005	0.0279	0.1562
Snowmelt date	−0.2347	0.122	0.054	−0.4733	0.0039
Snowmelt date^2^	0.2150	0.121	0.076	−0.0223	0.4523
Aspect (reference = North)					
East	−0.0082	0.036	0.818	−0.0785	0.0620
South	−0.0289	0.055	0.602	−0.1373	0.0795
West	−0.1850	0.034	<0.001	−0.2522	−0.1177
Flat	−0.2097	0.030	<0.001	−0.2687	−0.1507
Step length	−0.0003	<0.001	<0.001	−0.0003	−0.0003

Note

The forage variables included biomass (g/m^2^ dry mass [DM]), digestible nitrogen (g/100g DM), and digestible energy (kJ/g DM).

All patch scale top forage and mosquito models exhibited high predictive power based on k‐fold cross‐validation. The mean *r_s_* for the post‐calving, mosquito, and end of summer models were 0.94, 0.99, and 0.98, respectively, while for the forage*MI model it was 0.99. Note that cross‐validation was not conducted for the oestrid fly period models since forge variables did not significantly improve model fit.

### 3.3 Movement rates

**FIGURE 8:**
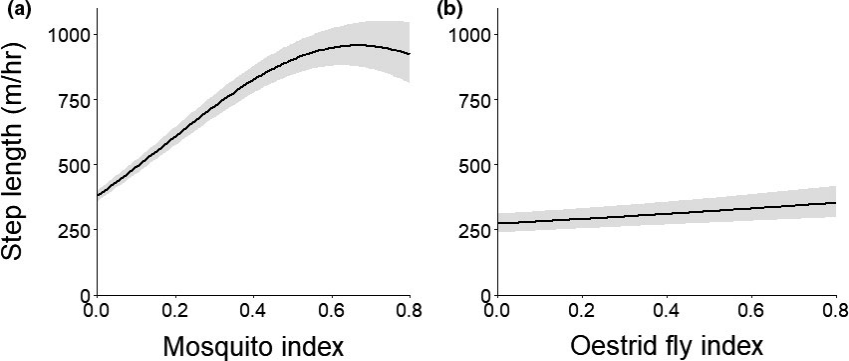
Step lengths (and 95% confidence intervals) of female caribou modeled as a function of the (a) mosquito and (b) oestrid fly activity indices during the mosquito (25 June–15 July) and oestrid fly (29 July–7 August) harassment periods, respectively, Central Arctic Herd, Alaska, 2015–2018. In each panel, the ordinal study date was held at its median value for each period

During the mosquito and oestrid fly periods, we found that greater insect activity increased caribou step lengths as hypothesized (Table 9). The top model for the mosquito period included linear and quadratic terms for MI and ordinal day, receiving 100% of the model weight (Table 9). As predicted, caribou substantially increased their movements in response to higher MI (Table 10), as their step lengths were ~2.5 times greater when MI was high compared with when it was low (Figure 8a). During the oestrid fly period, the top model included OI and a nonlinear response with ordinal day, garnering 55% of the model weight (Table 9), while the second best model captured the remaining weight and included a nonlinear response for OI. Caribou movements also increased in association with greater OI (Table 10), but the effect was relatively weak (Figure 8b). After accounting for insect activity, we found that caribou step lengths generally increased across the mosquito period, while they were relatively consistent during the oestrid fly period (Appendix S5).

**TABLE 9:** Model selection criteria for models of female caribou step lengths during the mosquito (25 June−15 July) and oestrid fly (29 July−7 August) harassment periods, Central Arctic Herd, Alaska, 2015–2018. Variables include the mosquito index (MI), oestrid fly index (OI), and study ordinal day (OD)

Period and Model	LL	AIC	ΔAIC	Weight
Mosquito harassment				
MI + MI^2^ + OD + OD^2^	−32,621.80	65,257.61	0.00	1.00
MI + OD + OD^2^	−32,671.50	65,355.01	97.40	0.00
MI + MI^2^ + OD	−32,686.08	65,384.15	126.54	0.00
MI + OD	−32,736.23	65,482.45	224.84	0.00
OD + OD^2^	−33,147.35	66,304.71	1,047.10	0.00
OD	−33,160.98	66,329.96	1,072.35	0.00
Oestrid fly harassment				
OI + OD + OD^2^	−12,356.77	24,725.54	0.00	0.55
OI + OI^2^ + OD + OD^2^	−12,355.99	24,725.98	0.44	0.44
OI + OI^2^ + OD	−12,363.97	24,739.93	14.39	0.00
OI + OD	−12,366.1	24,742.2	16.66	0.00
OD + OD^2^	−12,366.49	24,742.98	17.44	0.00
OD	−12,372.33	24,752.67	27.13	0.00

**TABLE 10:** Standardized coefficients from top models of female caribou step length during the mosquito (25 June–15 July) and oestrid fly (29 July–7 August) harassment periods in the Central Arctic Herd, Alaska, 2015–2018. The model for each period included the ordinal study day (OD), and either the mosquito index (MI) or oestrid fly index (OI)

Model and Coefficient	β	SE	L95% CI	U95% CI
Mosquito harassment				
Intercept	6.977	0.028	6.923	7.032
MI	0.401	0.014	0.373	0.428
MI^2^	−0.070	0.007	−0.084	−0.056
OD	0.239	0.009	0.221	0.256
OD^2^	−0.111	0.010	−0.130	−0.092
Oestrid fly harassment				
Intercept	6.332	0.068	6.199	6.464
OI	0.081	0.018	0.045	0.117
OD	−0.102	0.018	−0.137	−0.067
OD^2^	−0.100	0.023	−0.146	−0.055

## 4 DISCUSSION

Our findings elucidate the importance of spatiotemporal variation in forage for driving dynamic summer behavior of Arctic caribou and generally support our hypotheses that caribou selection of forage quantity and quality is temporally dynamic and scale dependent. At the landscape scale, during the early summer post‐calving and mosquito periods (when harassment was low), caribou selection met the predictions of the forage maturation hypothesis, as they selected areas with intermediate biomass and high forage quality (DN and DE). At the patch scale, however, caribou selected areas with both high forage biomass and quality, selecting for DN almost twice as strong as DE. During the early summer, this mixed strategy of selecting forage based on quality–quantity trade‐offs at broad spatial scales, but high quality and quantity at fine scales, likely enables caribou to maximize foraging opportunities, particularly for depositing body protein from high DN (Barboza et al., 2018). During the late summer, after DN declined below thresholds for storage, caribou behavior diverged and they generally selected areas with higher biomass and lower DN at both landscape and patch scales. We suspect that prioritizing areas with higher biomass enables caribou to gain mass for the coming winter (Chan‐McLeod et al., 1994). While we expected caribou to exhibit stronger selection for DE during late summer, our results likely reflect our finding that adequate forage DE was consistently available during this time (Figure 3). Our early and late summer selection results align with captive studies of caribou and reindeer that have found that protein deposition primarily occurs early in the growing season, and mass and fat deposition occurs later in the growing season (Chan‐McLeod et al., 1994, 1999). We suspect that the temporally dynamic, multi‐scaled foraging behavior we observed in the CAH may be shared across other populations of caribou and ungulates, particularly those in highly seasonal environments that have limited opportunities to accrue protein (McArt et al., 2009). The complexity of our findings likely also explains why past studies of foraging behavior in caribou and reindeer have yielded contradictory information, as results should be highly dependent upon the specific period and spatial scale investigated.

At both spatial scales, caribou selected forage components most strongly during early summer when the trade‐offs between forage quantity and quality were the greatest and exhibited high spatial variability. Caribou selection for high quality forage was enabled by their movements north across the Arctic Coastal Plain (Figure 1), following the receding snow and emergent plant growth (Severson et al., 2021; Skogland, 1980, 1984). Indeed, habitat used by caribou during the calving period had an average snowmelt date of 27 May, while habitat used further north during the post‐calving period had an average snowmelt date of 2 June, and habitat used even further north during the mosquito period had a snowmelt date of 5 June. Because proximity to the Arctic Ocean is associated with cooler temperatures and delayed phenology (Gustine et al., 2017), tracking early summer plant growth north across the coastal plain enabled caribou to maximize their exposure to high DN and DE. Although ungulates in temperate systems have been observed to “surf the green wave” during spring (Aikens et al., 2017; Merkle et al., 2016), CAH caribou appear to exhibit this behavior during early summer, given delayed phenology in the Arctic (Severson et al., 2021). During late summer, after DN declined below the threshold expected for protein gain, patterns of selection at both scales exhibited a stark switch, with caribou dispersing from the coast (Figure 1) into areas with generally higher biomass and DE (during the end of summer) and lower DN. It is important to note that although caribou appeared to avoid DN during late summer, we suspect this result was an artifact of higher biomass being negatively associated with DN. Caribou selection for forage during late summer was generally weaker than during early summer, and their distributions much more diffuse (Figure 1), likely because biomass and DE were sufficient across most of the CAH summer range (Figure 3). During the years of our study, the CAH was at moderate abundance (ADFG, 2020), so we do not expect that caribou density strongly influenced their access to forage quality or quantity.

Whereas caribou appeared to select equally for DN and DE at the landscape scale during early summer, our patch scale analyses revealed that the magnitude of selection for DN was nearly double that of DE, lending support to the hypothesis that Arctic caribou may be limited by forage protein (Barboza et al., 2018). For most of the year, caribou subsist on a diet that is poor in protein, consisting primarily of lichen and senesced vascular vegetation (Joly et al., 2015; Russell et al., 1993). As a result, their opportunity to amass nitrogen reserves is limited to early summer, nitrogen which is then conserved and used to produce a calf the following year (Barboza & Parker, 2006, 2008; Chan‐McLeod et al., 1994). High DN during early summer also coincides with the timing of peak lactation in caribou (Johnson et al., 2018; Klein, 1990), when the nitrogen costs for lactating females are ~120% above maintenance levels, whereas the energy costs are only ~50% above maintenance levels (Barboza & Parker, 2008). Given the importance of nitrogen to caribou reproduction and lactation, and its limited seasonal availability, we suspect that habitat selection that maximizes consumption of high forage DN is likely associated with fitness benefits, as has been found with high quality forage in other ungulate systems (Albon & Langvatn, 1992; Searle et al., 2015).

Mosquito activity significantly altered foraging opportunities for Arctic caribou, just as interspecific interactions with predators and competitors can reduce ungulate foraging opportunities in other systems (van Beest et al., 2013; Mason et al., 2014). At both spatial scales, caribou selected areas predicted to have reduced harassment, decreasing their exposure to mosquitos. For example, at the landscape scale, the median MI at caribou locations was only 0.07 while at available locations it was 0.27. As predicted, when caribou were subjected to higher MI values, they exhibited reduced selection for DN and DE (except at the landscape scale when forage values were very high). For example, at the patch scale, when the MI was low (≤0.05) the median DN at caribou locations was 1.28 g N/100 g DM, but when the MI was high (≥0.5) it was only 1.21 g N/100 g DM. At the landscape scale, mosquito harassment also fundamentally changed how caribou selected for biomass. In the absence of harassment, caribou selected for intermediate biomass in accordance with the forage maturation hypothesis, but when the MI increased, caribou selected areas with higher biomass as they shifted to drier vegetation types (similar to Walsh et al., 1992) with more advanced phenology, likely reducing their exposure to mosquitos. Importantly, this pattern differs from observations that CAH caribou use sparse vegetation (e.g., gravel bars) when mosquito harassment is high (White et al., 1975), areas which were excluded from our analyses because they were not sampled in the field.

As we predicted, higher MI values dramatically increased caribou movements, as they exhibited a ~2.5‐fold increase in their movement rate across the observed range of MI (Figure 8a). It has been assumed that increased mid‐summer movements are a function of insect harassment (Person et al., 2007; Joly et al., 2020), but our results provide empirical evidence of that relationship. Importantly, our results demonstrate that our index of mosquito activity influences caribou movements even at relatively low levels, with movement rates plateauing when the MI exceeds ≥0.6. The combined effects of mosquito harassment on forage selection and movement are particularly important given that they coincide with the short window of high DN, likely hindering caribou from acquiring protein during the limited time it is available. Assessing the effects of mosquito harassment on caribou body condition (Helle & Tarvainen, 1984) and demographic rates will become increasingly important as insect abundance and phenology respond to warmer Arctic conditions (Culler et al., 2015; Witter, Johnson, Croft, Gunn, & Gillingham, 2012). While we relied on a temperature‐ and wind‐based index of mosquito activity (Russell et al., 2013), we suspect that an index incorporating additional variables such as growing degree days and humidity would improve our ability to accurately predict harassment (Witter, Johnson, Croft, Gunn, & Poirier, 2012).

Although mosquito activity strongly influenced caribou habitat selection and movement behavior, oestrid fly activity appeared to have much less influence. Greater OI only weakly altered caribou selection of forage at the landscape scale, with caribou selecting areas with slightly less biomass. Although caribou movements increased with oestrid fly activity, the effect was minimal compared with mosquito activity (Figure 8). Caribou and reindeer have been observed to exhibit diverse behaviors in response to oestrid flies, increasing their time spent standing, walking, and running, while reducing time spent feeding (Hagemoen & Reimers, 2002; Mörschel & Klein, 1997; Toupin et al., 1996; Witter, Johnson, Croft, Gunn, & Gillingham, 2012). Perhaps these mixed responses contributed to our finding that movement rates were relatively similar for different levels of OI. Further, we suspect that the OI may be a relatively poor indicator of fly activity, as it was derived from the literature rather than field data (Russell et al., 1993). Interestingly, while the CAH responded more strongly to mosquito activity than oestrid fly activity, research on other caribou and reindeer populations has yielded opposite results. For example, the Bathurst caribou herd in Canada and reindeer in Norway both shifted their activity patterns strongly in response to oestrid flies but showed little response to mosquitos (Hagemoen & Reimers, 2002; Witter, Johnson, Croft, Gunn, & Gillingham, 2012). We suspect that the influence of different types of insects on caribou and reindeer is largely region‐specific, dependent upon the specific insect species present, their relative abundance, local weather conditions, and other site‐specific ecological conditions.

The window for Arctic caribou to amass DN appears to be narrow (Figure 2b; Barboza et al., 2018; Parrett, 2007) and changes in climate conditions threaten to reduce it further. For example, using forage data and models from the CAH, Johnson et al. (2018) found that years with earlier Arctic summer phenology were associated with reduced levels of predicted forage nitrogen during the summer. Similar patterns have been observed in fine‐scale studies of Arctic plants under experimental warming conditions, where warmer, earlier summer weather was associated with reduced plant nitrogen (Doiron et al., 2014; Jónsdóttir et al., 2005; Zamin et al., 2017). Additionally, if climate change results in earlier, more severe periods of mosquito harassment in the Arctic (Culler et al., 2015), harassment could begin during the post‐calving period, further depressing caribou use of high DN during the limited time it is available, while also increasing caribou movements. Fauchald et al. (2017) reported that caribou abundance across 11 Arctic populations was negatively correlated with warmer summer conditions and satellite measures of plant productivity. They suggested that the “greening of the Arctic” may be associated with reduced summer range quality for caribou. Although they were uncertain about the mechanism for such a relationship, we suspect that in years with earlier, warmer growing seasons, caribou may experience the compounding effects of reduced DN and increased insect harassment, which may have demographic consequences. It will be important to monitor summer forage and insect conditions, in association with caribou demographic rates, to assess support for these speculations.

In some caribou herds, the ability of animals to access high quality summer forage may also be inhibited by expanding human development. Movements of Arctic caribou to coastal habitat have been primarily attributed to seeking insect relief (White et al., 1975), but our work also highlights their importance in extending caribou access to high quality foraging areas. Coastal habitat used by caribou during the summer often overlaps with areas targeted for energy production (BLM, 2018, 2019a; Wilson et al., 2012), and caribou have been observed to avoid industrial development (Boulanger et al., 2012; Cameron et al., 2005; Johnson & Russell, 2014). For example, while the CAH will move through oil fields, Johnson et al. (2020) found that their use of habitat was less than expected within 5 km of energy development during the calving period, within 2 km during the post‐calving period, and within 1 km during the mosquito period. As a result, recent plans and proposals to expand energy development within the Arctic coastal plain (BLM, 2018; BLM, 2019a, 2019b,2019a, 2019b) could reduce caribou use of key habitat that provides both high quality forage and insect relief.

Our study elucidates important drivers of summer caribou behavior, but there were several limitations that are important to recognize. For example, we were not able to reliably evaluate foraging behavior during the calving period, which is of particular interest to wildlife managers and conservation practitioners. Caribou on the North Slope of Alaska birth calves in early June when there is still substantial snow cover, foraging on newly emergent, tussock cottongrass flowers along the receding snow edge (Eastland et al., 1989; Russell et al., 1993). Because our forage models were developed using snow‐free NDVI values, we were unable to predict forage components for large portions of the summer range during the calving period. This shortcoming prevented us from examining selection patterns during this key period, despite the assumption that forage quality is a primary determinant of the location of caribou calving grounds (Eastland et al., 1989; Griffith et al., 2002). Additionally, our modeled forage predictions were constrained to those coarse vegetation types that were sampled in the field. This area excluded 27% of the study area which was dominated by some vegetation types that may be important to caribou, notably low shrubs. Although CAH caribou avoid low shrub vegetation during early summer (Johnson et al., 2020), they likely increase their use of this vegetation type later in the summer (Wilson et al., 2012) as it may provide more energy than graminoid vegetation (Barboza et al., 2018). Missing nutritional information across portions of our study area could bias our results, and we recommend that future work aims to address this issue. Another limitation was that our models predicted average DN and DE for 6 key plant species, but caribou are known to be selective foragers (Denryter et al., 2017) that alter their diet across the summer (Parrett, 2007; Russell et al., 1993). Forage models that account for temporal shifts in diet composition would likely improve inferences about foraging behavior. Finally, given that field data on caribou forage conditions were only collected through August (Johnson et al., 2018), we were unable to predict temporal variation in forage components through the very end of the growing season (into September). If climate change is extending summer foraging opportunities later in the year, caribou may be able to amass more fat on their summer ranges (but see discussion about Arctic growing seasons in Myers‐Smith et al., 2020).

Our results demonstrate how early and late summer periods afford CAH caribou very distinct foraging opportunities, as changes in quality and quantity drive shifts in their distributions across the summer range. During early summer, female caribou select for high quality forage at broad landscape scales, and for both high quality and quantity forage at finer patch scales, a strategy that likely enables them to regain and amass protein stores. During late summer, caribou primarily select for high forage quantity at landscape and patch scales, a strategy that likely enables them to accrue mass and fat. These results highlight the importance of accounting for the period of the growing season, spatial scale, and the nutritional content of forage to elucidate the drivers of summer caribou behavior, factors which may similarly influence other ungulates, particularly capital breeders and those in nitrogen‐poor environments. Future research that links annual summer forage predictions to direct measures of caribou fitness (e.g., body condition, reproductive success, survival) will be important for validating our model‐based findings and assessing the resilience of caribou populations to variation in summer forage conditions.

## CONFLICT OF INTEREST

The authors declare no conflicts of interest.

## AUTHOR CONTRIBUTION

**Heather E. Johnson:** Conceptualization (lead); Data curation (equal); Formal analysis (lead); Writing‐original draft (lead); Writing‐review & editing (equal). **Trevor** **S Golden:** Data curation (equal); Formal analysis (supporting); Writing‐review & editing (equal). **Layne G. Adams:** Conceptualization (supporting); Funding acquisition (equal); Writing‐review & editing (equal). **David Gustine:** Conceptualization (supporting); Data curation (equal); Funding acquisition (equal). **Elizabeth A. Lenart:** Conceptualization (supporting); Data curation (equal); Funding acquisition (equal); Writing‐review & editing (equal). **Perry S. Barboza:** Conceptualization (supporting); Data curation (equal); Writing‐review & editing (equal).

## Supporting information

Appendix S1‐S5

## Data Availability

Forage data are available at https://doi.org/10.5066/F7JQ106W. Caribou locations are managed by Alaska Department of Fish and Game and are restricted from public access by Alaska Statue 16.05.815. All other data are available through the sources cited in the text.
