# Supplementary material for: Dynamic selection for forage quality and quantity in response to phenology and insects in an Arctic ungulate
Source: Ecol Evol. 2021 Aug 4;11(17):11664–88. doi: 10.1002/ece3.7852 (PMC8427565; doi:10.1002/ece3.7852)

**Appendix S1.** Modeling digestible nitrogen and digestible energy in summer caribou forage for the Central Arctic Herd, Alaska.

Following methods outlined in Johnson et al. (2018) and using data from Adams and Gustine (2018), we modeled field measurements of the average digestible nitrogen (DN; g/100g dry mass [DM]) and digestible energy (DE; kJ/g DM) of 6 key summer forage species for caribou in the Central Arctic Herd, Alaska. The forage species included tussock cottongrass (*Eriophorum vaginatum*), water sedge (*Carex aquatilis*), Bigelow's sedge (*Carex bigelowii*), Arctic lousewort (*Pedicularis* spp), diamond-leaf willow (*Salix pulchra*), and Richardson's willow (*Salix richardsonii*). We modeled variation in DN and DE using an all-subsets modeling approach with covariates including the satellite derived Normalized Difference Vegetation Index (NDVI) from eMODIS Alaska data (Jenkerson et al. 2010), the number of days before/after NDVI reached its maximum value for the summer (DaysToMax; 14 days before the maximum NDVI value = -14 while 5 days after the maximum value = 5), the distance to the coast (Coast; measured per 10 km), and the coarse vegetation type (Veg; categorical: tussock tundra, herbaceous mesic, herbaceous wet or dwarf shrub; Boggs et al. 2016). For each model, we calculated Akaike’s Information Criterion for small sample sizes (AIC_c_), ΔAIC_c_, and model weights (Table 1), and then we model-averaged parameter estimates for models with ΔAIC_c_ values < 10 (Table 2; Burnham and Anderson 2002). Models and coefficients of forage biomass (g/m^2^ DM) are reported in Johnson et al. (2018).

**Table 1**. Model selection criteria for high ranking models (ΔAIC_c_ < 10) of digestible nitrogen (g/100g dry mass [DM]) and digestible energy (kJ/g DM) of summer forage used by caribou in the Central Arctic Herd, Alaska. Model covariates included distance to the coast (Coast), the number of days before or after the Normalized Difference Vegetation Index (NDVI) reached its maximum summer value (DaysToMax), NDVI, and coarse vegetation type (Veg).

| Forage component and model | LL | AIC_c_ | ΔAIC_c_ | Weight |
| --- | --- | --- | --- | --- |
| Digestible Nitrogen |  |  |  |  |
| Coast+Coast^2^+DaysToMax+NDVI+NDVI^2^ | -11.17 | 39.34 | 0.00 | 0.25 |
| Coast+Coast^2^+DaysToMax | -13.58 | 39.73 | 0.40 | 0.20 |
| Coast+Coast^2^+DaysToMax+DaysToMax^2^+NDVI+NDVI^2^ | -10.47 | 40.19 | 0.85 | 0.16 |
| Coast+Coast^2^+DaysToMax+DaysToMax^2^ | -13.15 | 41.06 | 1.73 | 0.10 |
| Coast+Coast^2^+DaysToMax+NDVI | -13.50 | 41.77 | 2.43 | 0.07 |
| Coast+Coast^2^+DaysToMax+Veg | -11.93 | 43.10 | 3.76 | 0.04 |
| Coast+Coast^2^+DaysToMax+NDVI+NDVI^2^+Veg | -9.67 | 43.19 | 3.86 | 0.04 |
| Coast+Coast^2^+DaysToMax+DaysToMax^2^+NDVI | -13.15 | 43.29 | 3.95 | 0.03 |
| Coast+Coast^2^+DaysToMax+DaysToMax^2^+NDVI+NDVI^2^+Veg | -9.02 | 44.23 | 4.90 | 0.02 |
| Coast+Coast^2^+DaysToMax+DaysToMax^2^+Veg | -11.48 | 44.50 | 5.16 | 0.02 |
| Coast+Coast^2^+DaysToMax+NDVI+Veg | -11.81 | 45.16 | 5.82 | 0.01 |
| DaysToMax | -18.84 | 45.94 | 6.61 | 0.01 |
| DaysToMax+NDVI+NDVI^2^ | -16.73 | 46.02 | 6.68 | 0.01 |
| DaysToMax+DaysToMax^2^+NDVI+NDVI^2^ | -15.97 | 46.70 | 7.36 | 0.01 |
| Coast+Coast^2^+DaysToMax+DaysToMax^2^+NDVI+Veg | -11.48 | 46.80 | 7.47 | 0.01 |
| DaysToMax+DaysToMax^2^ | -18.44 | 47.29 | 7.95 | 0.01 |
| DaysToMax+NDVI | -18.83 | 48.07 | 8.73 | 0.00 |
| Coast+DaysToMax | -18.83 | 48.07 | 8.73 | 0.00 |
| Coast+DaysToMax+NDVI+NDVI^2^ | -16.72 | 48.19 | 8.86 | 0.00 |
| Coast+DaysToMax+DaysToMax^2^+NDVI+NDVI^2^ | -15.96 | 48.92 | 9.58 | 0.00 |
| Digestible Energy |  |  |  |  |
| Coast+Coast^2^+NDVI+NDVI^2^+Veg | -310.55 | 642.63 | 0.00 | 0.18 |
| Coast+Coast^2^+NDVI+Veg | -311.77 | 642.79 | 0.16 | 0.17 |
| Coast+Coast^2^+DaysToMax+NDVI+NDVI^2^+Veg | -309.92 | 643.69 | 1.07 | 0.11 |
| Coast+Coast^2^+DaysToMax+NDVI+Veg | -311.46 | 644.45 | 1.82 | 0.07 |
| Coast+NDVI+Veg | -313.78 | 644.55 | 1.92 | 0.07 |
| Coast+NDVI+NDVI^2^+Veg | -312.79 | 644.82 | 2.20 | 0.06 |
| Coast+Coast^2^+DaysToMax+DaysToMax^2^+NDVI+NDVI^2^+Veg | -309.40 | 645.01 | 2.38 | 0.06 |
| Coast+DaysToMax+NDVI+NDVI^2^+Veg | -311.76 | 645.05 | 2.42 | 0.05 |
| Coast+DaysToMax+NDVI+Veg | -313.16 | 645.55 | 2.93 | 0.04 |
| Coast+DaysToMax+DaysToMax^2^+NDVI+NDVI^2^+Veg | -311.15 | 646.14 | 3.51 | 0.03 |
| Coast+Coast^2^+DaysToMax+DaysToMax^2^+NDVI+Veg | -311.18 | 646.21 | 3.59 | 0.03 |
| Coast+DaysToMax+DaysToMax^2^+NDVI+Veg | -312.80 | 647.13 | 4.50 | 0.02 |
| Coast+Coast^2^+DaysToMax+DaysToMax^2^+Veg | -312.81 | 647.15 | 4.53 | 0.02 |
| Coast+DaysToMax+DaysToMax^2^+Veg | -313.97 | 647.18 | 4.55 | 0.02 |
| Coast+DaysToMax+Veg | -315.21 | 647.41 | 4.78 | 0.02 |
| Coast+Coast^2^+DaysToMax+Veg | -314.09 | 647.41 | 4.79 | 0.02 |
| DaysToMax+Veg | -316.39 | 647.54 | 4.92 | 0.02 |
| DaysToMax+DaysToMax^2^+Veg | -315.31 | 647.61 | 4.99 | 0.02 |
| Coast+Coast^2^+Veg | -316.08 | 649.15 | 6.52 | 0.01 |
| Coast+Veg | -317.59 | 649.94 | 7.31 | 0.01 |
| Veg | -318.81 | 650.20 | 7.57 | 0.00 |

**Table 2.** Model-averaged parameter estimates predicting the average digestible nitrogen (g/100g dry mass [DM]) and digestible energy (kJ/g DM) of summer forage used by caribou in the Central Arctic Herd, Alaska. Model covariates included the Normalized Difference Vegetation Index (NDVI), the number of days before or after NDVI reached its maximum summer value (DaysToMax), distance to the coast (Coast), and coarse vegetation type. The reference class for vegetation type was tussock tundra. Averaging was conducted from models presented in Table 1 (this appendix), which included those with ΔAIC_c_ < 10.

| Forage component and coefficient | β | SE | L95% CI | U95% CI |
| --- | --- | --- | --- | --- |
| Digestible Nitrogen |  |  |  |  |
| Intercept | 0.99511 | 0.25978 | 0.48594 | 1.50427 |
| NDVI | 1.34445 | 0.96029 | -0.53769 | 3.22659 |
| NDVI^2^ | -1.61292 | 0.73482 | -3.05313 | -0.17270 |
| DaysToMax | -0.00838 | 0.00082 | -0.00999 | -0.00676 |
| DaysToMax ^2^ | -0.00002 | 0.00002 | -0.00007 | 0.00002 |
| Coast | -0.05215 | 0.01563 | -0.08279 | -0.02152 |
| Coast^2^ | 0.00267 | 0.00071 | 0.00129 | 0.00406 |
| Vegetation type (reference = Tussock tundra) |  |  |  |  |
| Dwarf shrub | 0.02726 | 0.09826 | -0.16532 | 0.21984 |
| Herbaceous mesic | -0.06264 | 0.09283 | -0.24458 | 0.11930 |
| Herbaceous wet | -0.12868 | 0.10685 | -0.33810 | 0.08074 |
| Digestible Energy |  |  |  |  |
| Intercept | 7.19976 | 1.94066 | 3.39615 | 11.00338 |
| NDVI | 8.10261 | 5.87111 | -3.40456 | 19.60979 |
| NDVI^2^ | -8.28502 | 5.09741 | -18.27576 | 1.70573 |
| DaysToMax | 0.00614 | 0.00596 | -0.00555 | 0.01783 |
| DaysToMax ^2^ | -0.00018 | 0.00017 | -0.00051 | 0.00015 |
| Coast | -0.06189 | 0.13135 | -0.31932 | 0.19554 |
| Coast^2^ | 0.00976 | 0.00506 | -0.00016 | 0.01968 |
| Vegetation type |  |  |  |  |
| Dwarf shrub | 3.63165 | 0.71123 | 2.23765 | 5.02564 |
| Herbaceous mesic | 1.34887 | 0.65604 | 0.06306 | 2.63467 |
| Herbaceous wet | 2.79961 | 0.76038 | 1.30930 | 4.28992 |

References

Adams, L. G., and D. D. Gustine. 2018. Caribou forage and soil data, North Slope of Alaska,

2011-2014. U.S. Geological Survey data release: <https://doi.org/10.5066/F7JQ106W>.

Boggs, K., L. Flagstad, T. Boucher, T. Kuo, D. Fehringer, S. Guyer, and M. Aisu. 2016.

Vegetation map and classification: Northern, Western, and Interior Alaska. Second edition. Alaska Center for Conservation Science, University of Alaska Anchorage, Anchorage, Alaska, USA.

Burnham, K. P., and D. R. Anderson. 2002. Model selection and multimodel inference: a

practical information-theoretic approach. Second edition. Springer, New York, New York, USA.

Jenkerson, C. B., T. Maiersperger, and G. L. Schmidt. 2010. eMODIS: a user-friendly data

source. U.S. Geological Survey Open File Report 2010-1055, Reston, Virginia, USA.

Johnson, H. E., D. D Gustine, T. S. Golden, L. G. Adams, L. S. Parrett, E. A. Lenart, and P. S.

Barboza. 2018. NDVI exhibits mixed success in predicting spatiotemporal variation in caribou summer forage quality and quantity. Ecosphere 9:e02461.

**Appendix S2.** The means and ranges of continuous variables included in landscape and patch scale models of summer female caribou habitat selection, Central Arctic Herd, Alaska, 2015-2018. Variable values are provided for used and available locations and included biomass (g/m^2^ dry mass [DM]), digestible nitrogen (DN; g/100g DM), digestible energy (DE; kJ/g DM), elevation (m), snowmelt date (ordinal day), the mosquito harassment index (MI), and the oestrid fly harassment index (OI).

|  |  |  | Used Locations | |  | Available Locations | |
| --- | --- | --- | --- | --- | --- | --- | --- |
| Scale and Period | Covariate |  | Mean | Range |  | Mean | Range |
| Landscape Scale |  |  |  |  |  |  |  |
| Post-calving | Biomass |  | 18.49 | 0.00 - 83.30 |  | 25.23 | 0.00 - 111.07 |
|  | DN |  | 1.31 | 0.72 - 1.72 |  | 1.27 | 0.00 - 2.00 |
|  | DE |  | 10.06 | 6.16 - 12.65 |  | 10.06 | 4.88 - 15.84 |
|  | Elevation |  | 73.67 | 1.00 - 527.17 |  | 283.11 | 1.00 - 1819.54 |
|  | Snowmelt date |  | 152.6 | 129.0 - 210.0 |  | 151.4 | 55.0 - 213.0 |
| Mosquito harassment | Biomass |  | 27.01 | 0.00 - 102.92 |  | 35.53 | 0.00 - 113.86 |
|  | DN |  | 1.25 | 0.43 - 1.74 |  | 1.14 | 0.00 - 1.96 |
|  | DE |  | 10.55 | 6.80 - 12.76 |  | 10.23 | 3.86 - 15.85 |
|  | Elevation |  | 36.86 | 1.00 - 488.45 |  | 283.35 | 1.00 - 1947.05 |
|  | Snowmelt date |  | 156.1 | 112.0 - 212.0 |  | 153.3 | 58.0 - 213.0 |
|  | MI |  | 0.14 | 1.00 - 0.97 |  | 0.27 | 0.00 - 0.99 |
| Oestrid fly harassment | Biomass |  | 44.11 | 0.00 - 97.95 |  | 44.13 | 0.00 - 113.33 |
|  | DN |  | 0.83 | 0.00 - 1.56 |  | 0.86 | 0.00 - 1.83 |
|  | DE |  | 10.70 | 4.07 - 15.64 |  | 10.41 | 2.71 - 15.81 |
|  | Elevation |  | 526.70 | 1.00 - 1389.10 |  | 293.28 | 1.00 - 1840.73 |
|  | Snowmelt date |  | 152.5 | 112.0 - 212.0 |  | 151.0 | 58.0 - 213.0 |
|  | OI |  | 0.14 | 0.00 - 0.99 |  | 0.14 | 0.00 - 0.99 |
| End of summer | Biomass |  | 46.19 | 1.00 - 113.34 |  | 37.11 | 0.00 - 114.27 |
|  | DN |  | 0.68 | 0.00 - 1.47 |  | 0.71 | 0.00 - 1.70 |
|  | DE |  | 9.77 | 0.00 - 14.93 |  | 10.36 | 1.56 - 15.81 |
|  | Elevation |  | 241.20 | 4.00 - 1405.60 |  | 286.80 | 1.00 - 1881.61 |
|  | Snowmelt date |  | 151.8 | 113.0 - 212.0 |  | 153.2 | 61.0 - 213.0 |
|  |  |  |  |  |  |  |  |
| Patch Scale |  |  |  |  |  |  |  |
| Post-calving | Biomass |  | 18.46 | 0.00 - 83.30 |  | 17.86 | 0.00 - 83.30 |
|  | DN |  | 1.31 | 0.72 - 1.72 |  | 1.31 | 0.00 - 1.76 |
|  | DE |  | 10.06 | 6.16 - 12.65 |  | 10.09 | 5.14 - 12.74 |
|  | Elevation |  | 73.05 | 1.00 - 527.17 |  | 73.64 | 1.00 - 1015.75 |
|  | Snowmelt date |  | 152.6 | 129.0 - 210.0 |  | 152.9 | 119.0 - 212.0 |
| Mosquito harassment | Biomass |  | 27.08 | 0.00 - 102.92 |  | 25.78 | 0.00 - 113.83 |
|  | DN |  | 1.25 | 0.43 - 1.74 |  | 1.24 | 0.00 - 1.78 |
|  | DE |  | 10.55 | 6.80 - 12.76 |  | 10.63 | 3.34 - 12.78 |
|  | Elevation |  | 36.85 | 1.00 - 488.45 |  | 37.81 | 1.00 - 1011.78 |
|  | Snowmelt date |  | 156.0 | 112.0 - 212.0 |  | 156.4 | 111.0 - 212.0 |
|  | MI |  | 0.137 | 0.000 - 0.953 |  | 0.142 | 0.00 - 0.967 |
| Oestrid fly harassment | Biomass |  | 44.23 | 0.00 - 97.95 |  | 44.44 | 0.00 - 106.26 |
|  | DN |  | 0.83 | 0.00 - 1.56 |  | 0.83 | 0.00 - 1.70 |
|  | DE |  | 10.70 | 4.07 - 15.64 |  | 10.62 | 1.61 - 15.93 |
|  | Elevation |  | 527.40 | 1.00 - 1389.10 |  | 514.50 | 1.00 - 1668.70 |
|  | Snowmelt date |  | 152.4 | 112.0 - 212.0 |  | 152.3 | 61.0 - 213.0 |
|  | OI |  | 0.141 | 0.00 - 0.988 |  | 0.140 | 0.00 - 0.993 |
| End of summer | Biomass |  | 46.19 | 0.00 - 113.34 |  | 45.39 | 0.00 - 113.34 |
|  | DN |  | 0.68 | 0.00 - 1.47 |  | 0.68 | 0.00 - 1.48 |
|  | DE |  | 9.76 | 0.00 - 14.93 |  | 9.80 | 0.83 - 15.65 |
|  | Elevation |  | 239.80 | 4.00 - 1405.60 |  | 238.60 | 2.00 - 1523.70 |
|  | Snowmelt date |  | 151.7 | 113.0 - 212.0 |  | 151.7 | 89.0 - 213.0 |
|  |  |  |  |  |  |  |  |

**Appendix S3.** Proportion of female caribou locations considered to be snow-free across the summer as estimated by Moderate Resolution Imaging Spectroradiometer satellite imagery, Central Arctic Herd, Alaska, 2015-2018. Locations are color coded by whether they were obtained during the calving (1−15 Jun), post-calving (16−24 Jun), mosquito harassment (25 Jun−15 Jul), oestrid fly harassment (29 Jul−7 Aug) or end of summer (16−31 Aug) periods. Note that much of the study area was covered in snow during the calving period, and that the entire study area was snow-free by the oestrid fly harassment period.


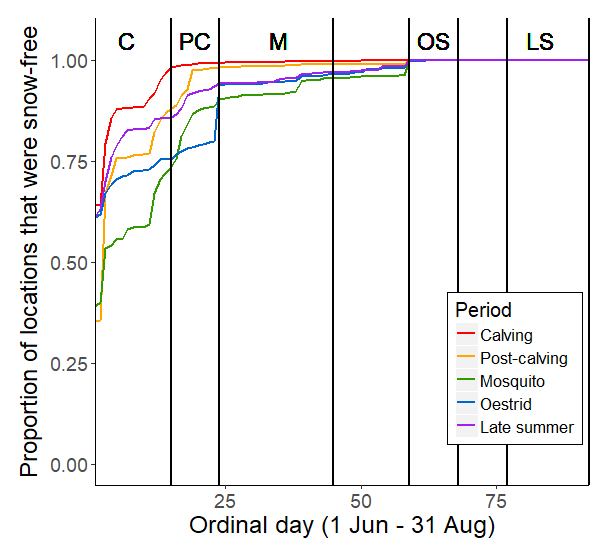


**Appendix S4.** Landscape scale relative probability of selection (and 95% confidence interval) of female caribou for areas with different levels of the oestrid fly activity index during the oestrid fly harassment period (29 Jul−7 Aug), Central Arctic Herd, Alaska, 2015-2018. Probabilities were derived from the top model while holding all other covariates at their mean values for used locations.


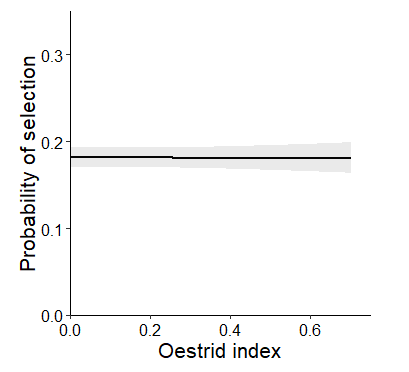


**Appendix S5**. Step length (and 95% confidence interval) of female caribou as a function of the study day during the (a) mosquito harassment period (25 Jun−15 Jul; days 25−45) and (b) oestrid fly harassment period (29 Jul−7 Aug; days 59−68), Central Arctic Herd, Alaska, 2015-2018.


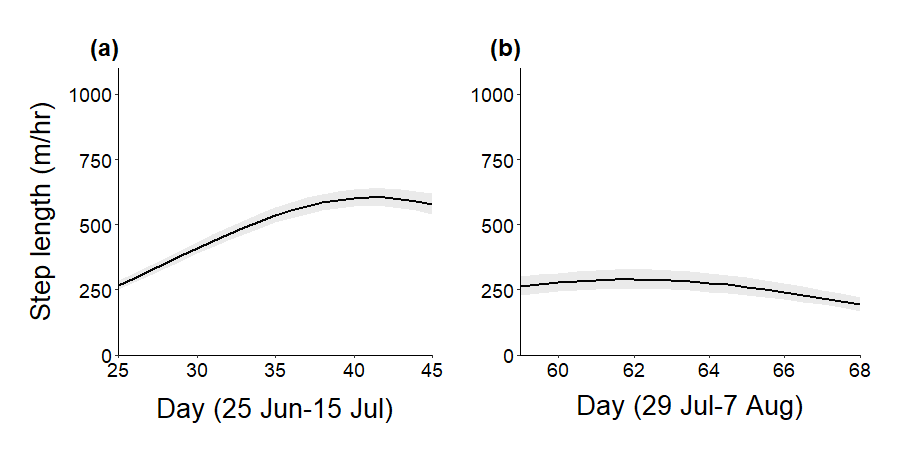

Supplement: Supplementary file 1 — Appendix S1‐S5 [file ECE3-11-11664-s001.docx]
